# Myelofibrosis management in routine clinical practice with a focus on patients with cytopenias: recommendations from a global consensus group

**DOI:** 10.1038/s41375-024-02330-7

**Published:** 2024-07-09

**Authors:** Steffen Koschmieder, Prithviraj Bose, Martin H. Ellis, Vikas Gupta, Jean-Jacques Kiladjian, John Mascarenhas, Vikram Mathews, Francesco Passamonti, Claire Harrison

**Affiliations:** 1 Department of Hematology, Oncology, Hemostaseology, and Stem Cell Transplantation, Faculty of Medicine, RWTH Aachen University, and Center for Integrated Oncology Aachen Bonn Cologne Düsseldorf (CIO ABCD), Aachen, Germany; 2 The University of Texas MD Anderson Cancer Center, Houston, TX USA; 3 Hematology Institute and Blood Bank, Meir Medical Center and Faculty of Medicine, Tel Aviv University, Tel Aviv, Israel; 4 Princess Margaret Cancer Centre, University of Toronto, Toronto, ON Canada; 5 Hôpital Saint-Louis, Université Paris Cité, Inserm CIC-1427, Paris, France; 6 Tisch Cancer Institute, Icahn School of Medicine at Mount Sinai, New York, NY USA; 7 Department of Haematology, Christian Medical College, Vellore, Tamil Nadu India; 8 Università degli Studi di Milano, Fondazione IRCCS Ca’ Granda Ospedale Maggiore Policlinico, Milano, Italy; 9 Department of Haematology, Guy’s and St Thomas’ NHS Foundation Trust, London, UK

**Keywords:** Haematological cancer, Cancer

## To the Editor:

Myelofibrosis (MF) is a Philadelphia chromosome (*BCR::ABL1*)-negative myeloproliferative neoplasm, a hallmark of which is progressive deposition of fibrotic tissue in bone marrow [1]. Clinical manifestations of MF often include splenomegaly, cytopenias (such as severe anemia), and extramedullary hematopoiesis [1]. The Janus kinase inhibitor (JAKi) therapies ruxolitinib (RUX) and fedratinib (FED) have demonstrated significant clinical efficacy in splenic volume reduction and symptom improvement, but they may induce treatment-related anemia and thrombocytopenia [2–9]. Other JAKi options include pacritinib (PAC), which received FDA approval in 2022 for patients with MF and severe thrombocytopenia (platelet count <50 × 10^9^/l), and momelotinib (MMB), which received FDA and EMA approval in 2023/2024, respectively, for patients with MF and anemia [10–12]. Clinical trials with JAKis in MF are summarized in reference [1].

National and international guidelines exist for the management of MF; however, a need remains for practical guidance applicable in everyday clinical practice, especially for patients experiencing cytopenias or potential failure of current therapy. The landscape is further complicated by the availability of multiple prognostic tools for MF; as such, clinicians may find disease prognostication challenging and confusing. Additionally, to maximize clinical applicability of trial data, inclusivity of eligibility criteria in the context of the real-world MF patient population should be considered.

Recognizing these significant challenges, an international expert consensus group was established to provide best practice recommendations for healthcare professionals, intending to supplement, but not replace, existing guidelines.

The consensus program was conducted between March and September 2023, led by a Steering Committee (SC) of nine international hematology experts. The program utilized a modified Delphi methodology; the SC performed several rounds of consensus statement review before submission to the Extended Faculty (EF) of hematologists and patients—nominated by the SC—for a decisive vote. Funding was provided by GlaxoSmithKline (GSK), Brentford, UK. Importantly, GSK had no involvement in formulating questions or recommendations, and no representative participated in the voting process. The SC identified 25 key clinical questions across five consensus themes:

1. Defining the thresholds for anemia, and when to initiate/modify treatment
2. Defining the threshold for thrombocytopenia and when to initiate/modify treatment
3. Defining JAKi failure and what would warrant switching treatment
4. How and when to determine prognosis in patients with MF
5. Unmet needs in MF clinical trials

The EF assessed the importance of the questions, resulting in the selection of the 15 highest scoring questions for the consensus program. To address each, a systematic literature review was conducted following the PICO (Population, Intervention, Comparison, Outcome) framework, providing a comprehensive evaluation of relevant evidence (see Supplementary Table S1). From this, the SC developed a clinical recommendation (CR) to address each question. An online voting platform was used to obtain agreement scores for each recommendation from the SC (nine hematologists) and EF (20 hematologists and nine patients). Consensus was achieved when ≥75% of respondents agreed within the range of 7–9 on a 9-point scale (1=strongly disagree, 9=strongly agree). The strength of each recommendation was identified by obtaining the median and mean values from the votes (see Table 1). The level of consensus was determined by the percentage of votes falling within the range of 7–9 among all obtained votes.

**Table 1:** Consensus recommendations.

**Theme 1: Defining the Thresholds for Anemia, and When to Initiate/Modify Treatment**				
**Q1. What is the appropriate workup for anemia diagnosis in a patient with MF?**				
**Consensus recommendation**			**Strength of recommendation,**^**a**^ **median score (mean score)**	**Level of consensus**^**b**^
***CR1:*** *Anemia in MF is frequently multifactorial; workup should include evaluation of iron/vitamin B*_*12*_*/folate levels, exclusion of hemolysis and active bleeding, assessment for disease progression and any other comorbidity (see table below), and exclusion of treatment effect. In cases of hemolysis, additional investigations are recommended*. **Summary of Diagnostic Tests for Anemia Workup in Patients with MF**			8 (8.22)	95.65% n/N = 23/38
**Primary diagnostic workup**	**Diagnostic test**	**Additional information**		
**Initial workup**	**CBC, reticulocytes, differential (blood film)**	• Mandatory to decide on second line of investigation, based on Hb, MCV, and RDW; review disease progression, e.g., blasts (if these are detected, repeat karyotype, genetics, and BM biopsy may be needed)		
	**EPO level**	–		
	**Iron/vitamin B**_**12**_**/folate levels**	–		
	**Renal profile**	–		
**Exclude active bleeding**	**Stool and urine testing**, **imaging, or endoscopy, gynecologic examination**	–		
**Exclude hemolytic anemia**	**Peripheral blood film** **and reticulocyte count**	• Abnormalities of RBC morphology often suggest the presence of hemolysis, but are difficult to evaluate with concurrent MF • Consider PNH		
	**Serum bilirubin, haptoglobin, and ALT levels**	• Elevated indirect bilirubin with normal ALT • LDH is not interpretable in the context of MF		
	**Coombs test**	• Both direct and indirect Coombs testing should be performed, including testing for the presence of C3d. If the patient has received a transfusion in the last 3 months, a positive result could also indicate alloantibodies to transfused RBCs		
**Exclude hemoglobinopathies**	**Hb electrophoresis**	• To be performed if indicated		
*ALT, alanine transaminase; BM, bone marrow; C3, complement component 3; CBC, complete blood count; EPO, erythropoietin; Hb, hemoglobin; LDH, lactate dehydrogenase; MCV, mean corpuscular volume; MF, myelofibrosis; PNH, paroxysmal nocturnal hemoglobinuria; RBC, red blood cell; RDW, red cell distribution width*.				
**Q2. When should treatment (that is not transfusion based) be initiated/modified to improve anemia? Which patient characteristics should be considered?**				
***CR2:*** *Treatment for anemia (that is not transfusion based) should be considered for patients with a hemoglobin (Hb) level of* <*10* *g/dl, and in some cases at higher Hb levels; for example, anemia following initiation of therapy (e.g., Janus kinase [JAK] inhibitor) should be anticipated and therapy dose should be optimized. For persistent anemia, consider addition of a treatment, such as erythropoietin-stimulating agents (ESAs) for patients with erythropoietin (EPO) levels* <*500 IU, or treatment with an agent such as danazol or luspatercept that abrogates anemia, if EPO levels are* > *500IU or the patient is refractory to ESAs*. *Use of these agents is currently off-label in this setting*.			8 (8.04)	91.30% n/N = 23/38
**Q3. Which current and emerging treatments to improve anemia should be considered for:** • **MF-related anemia?** • **Treatment-related anemia?**				
***CR3:*** *Once other causes such as disease progression have been excluded: for MF-related anemia, Janus kinase (JAK) inhibition with momelotinib or pacritinib, danazol, luspatercept, erythropoietin-stimulating agents (ESAs), immunomodulatory drugs (IMiD*^*®*^*), or conventional combination therapies, such as JAK inhibition plus ESAs, danazol, luspatercept, or IMiD*^*®*^, may overcome the necessity of dose adjustments/interruptions, which may be associated with ruxolitinib or fedratinib.* In the future, novel combination therapies may deliver these benefits. Splenectomy can be considered as a last resort in select cases of refractory disease-related anemia. For treatment-related anemia, consider dose reduction of current therapy for 4–6 weeks. *Only ruxolitinib, fedratinib, pacritinib, and momelotinib are approved treatments for MF.			8 (8.22)	100% n/N = 23/38
**Q4. Aside from access and reimbursement, what factors guide selection of JAK inhibitor therapy in patients with MF and anemia?**				
***CR4:*** *Factors guiding selection of Janus kinase (JAK) inhibitor monotherapy would include:* • *Baseline hemoglobin (Hb)* • *Likely tolerance of anemia* • *Baseline thrombocytopenia* • *MF-related symptoms* *For some agents, consideration of drug-specific adverse events (immunosuppression, skin cancer, infection risk, nutritional status, tolerance of gastrointestinal [GI] toxicity, neurotoxicity, cardiovascular adverse events) is also a factor*.			8.5 (8.17)	91.67% n/N = 24/38
**Theme 2: Defining the Threshold for Thrombocytopenia and When to Initiate/Modify Treatment**				
**Q5. Which treatments for MF can be safely administered to patients with thrombocytopenia, and when should treatment be initiated/modified?**				
**Consensus recommendation**			**Strength of recommendation**,^**a**^ **median score (mean score)**	**Level of consensus**^**b**^
***CR5:***^***c***^ *Management of spleen, symptoms, and anemia in patients with MF and a platelet count of 50–100×10*^*9*^*/l with pacritinib, fedratinib, momelotinib, or low-dose ruxolitinib is feasible. Management in patients with MF and a platelet count of* <*50×10*^*9*^*/l is complex. Pacritinib is approved by the US Food and Drug Administration for patients with a platelet count of* <*50×10*^*9*^*/l, and there are reports that the use of fedratinib, momelotinib, or low-dose ruxolitinib may be feasible in this setting. It is important to consider the risk of bleeding associated with thrombocytopenia and concomitant use of anticoagulation/antiplatelet therapy, and, possibly, consider prophylaxis with antifibrinolytics*.			8 (8.17)	95.65% n/N = 23/38
**Q6. Which treatments to increase platelet count can be safely administered to patients with MF and thrombocytopenia, and when should treatment be initiated/modified?**				
***CR6:*** *Treatments to increase platelet counts are rarely effective; agents such as low-dose corticosteroids, danazol, or low-dose immunomodulatory drugs (IMiD*^*®*^*) could be considered. There are no data supporting the safety or benefit of thrombopoietin (TPO) mimetics in this setting. Splenectomy can be considered as a last resort in select cases*.			8 (7.96)	91.30% n/N = 23/38
**Q7. Aside from access and reimbursement, what factors guide selection of JAK inhibitor therapy in patients with MF and thrombocytopenia?**				
***CR7:***^***c***^ *Factors guiding selection of Janus kinase (JAK) inhibitor monotherapy would include:* • *Baseline hemoglobin (Hb)* • *Degree of thrombocytopenia (e.g., the only currently approved therapy for patients with platelets* *<50×10*^*9*^*/l* *is pacritinib)* • *MF-related symptoms* *For some agents, consideration of drug-specific adverse events (immunosuppression, skin cancer, infection risk, nutritional status, tolerance of gastrointestinal [GI] toxicity, neurotoxicity, cardiovascular adverse events) is also a factor*.			9 (8.25)	91.67% n/N = 24/38
**Theme 3: Defining JAK Inhibitor Failure and What Would Warrant Switching Treatment**				
**Q8. What criteria should be used to define a patient who is relapsed, refractory, or intolerant to JAK inhibitor treatment?**				
**Consensus recommendation**			**Strength of recommendation**,^**a**^ **median score (mean score)**	**Level of consensus**^**b**^
***CR8:*** *There are existing criteria for ruxolitinib that are used in clinical trials to determine if a patient is relapsed, refractory, or intolerant to treatment (see* Supplementary Table S10*); however, in clinical practice, it may be difficult to distinguish exactly between ruxolitinib intolerance and relapse, as often these can coexist. Criteria for other Janus kinase (JAK) inhibitors are likely to be similar*.			8 (8.09)	94.12% n/N = 34/38
**Q9. How is a suboptimal JAK inhibitor response defined?**				
***CR9:*** *Suboptimal response to a Janus kinase (JAK) inhibitor could be defined as instances where the JAK inhibitor retains benefit in some aspects of disease but not others. For example, residual splenomegaly or symptoms, or failure to achieve an anemia response, where this is the target of therapy with drugs such as momelotinib or pacritinib.(See* Supplementary Table S10*for criteria for ruxolitinib failure used in the re-analysis of the JAKARTA-2, PAC203, and FREEDOM trials)*.			8 (7.8)	85.71% n/N = 35/38
**Q10. Which MF parameters should be incorporated into response assessments? When should they be repeated and how often?**				
***CR10:*** *Response assessments should be repeated every 3–6 months, depending on MF risk category and stability, and include:* • *Accurately determined spleen size (e.g., using a measuring tape)* • *Symptom score with a validated tool such as the Myeloproliferative Neoplasm Symptom Assessment Form Total Symptom Score (MPN-SAF TSS) or the Myelofibrosis Symptom Assessment Form (MFSAF)* • *Full blood count, including circulating blast percentage* • *Control of elevated blood counts* • *Degree of anemia and/or thrombocytopenia* • *Formalized prognostic risk assessment of disease in patients of transplant-eligible age* *There are no data for how often next-generation sequencing (NGS) or karyotyping should be performed, but alongside a repeated bone marrow biopsy, this should be considered if disease progression is suspected, warranting discontinuation of therapy or changing line of therapy. Other biomarkers such as cytokine levels, changes in bone marrow fibrosis grade, or variant allele burden assessment are investigational. Monitoring of driver mutation variant allele frequency (VAF) is not currently recommended but may be important in the future*.			8 (8.14)	88.89% n/N = 36/38
**Q11. Which clinical characteristics determine when a treatment switch is warranted?**				
***CR11:*** *Treatment should be given at a maximum tolerated dose for 3–6 months to determine best response. A treatment switch is warranted/should be considered in accordance with previous definitions for relapsed, refractory, suboptimal response, or a lack of maintained response. For intolerance or toxicity, it should be noted that criteria may be reached sooner. Treating physicians should be cautious of discontinuation syndrome (best described with ruxolitinib)*.			9 (8.31)	97.22% n/N = 36/38
**Theme 4: How and When to Determine Prognosis in Patients With MF**				
**Q12. Which prognostic scoring tools should be used:** • **For patients with pre-PMF?** • **At diagnosis?** • **During the course of MF disease?** • **To determine transplantation risk?**				
**Consensus recommendation**			**Strength of recommendation**,^**a**^ **median score (mean score)**	**Level of consensus**^**b**^
***CR12:*** Supplementary Table S11*contains prognostic scores for primary MF (PMF) or post-polycythemia vera/-essential thrombocytopenia (PV/-ET) MF that have been validated for use at diagnosis, during the course of disease, and to determine transplantation risk. It is important to be aware of the limitations and appropriate use of each of these scores. The development of prognostic scores for patients with pre-PMF is an unmet need; however, the International Prognostic Scoring System (IPSS) has been validated in this setting*.			9 (8.26)	97.14% n/N = 35/38
**Q13. Which clinical variables are predictive of long-term outcome or survival benefit in patients receiving JAK inhibitor therapy?**				
***CR13:*** *The following variables may be useful in predicting the outcome for patients receiving Janus kinase (JAK) inhibitor therapy; however, it is important to consider the limitations of survival data and the fact that these variables are yet to be validated in large datasets:* • *Spleen size reduction (for ruxolitinib, pacritinib, momelotinib, and fedratinib)* • *Mutational complexity (for ruxolitinib)* • *Transfusion independence (for momelotinib)* • *Requirement for transfusion and dose of ruxolitinib* *<20* *mg BID (for patients who have been on RUX for* *≥6 months) – in line with the RR6 prognostic score* *Emergence of clonal progression (following discontinuation of ruxolitinib) and specifically, clones such as RAS and TP53.*			8 (8.09)	100% n/N = 23/38
**Theme 5: Unmet Needs in MF Clinical Trials**				
**Q14. How can broader inclusion in clinical trials be achieved?**				
**Consensus recommendation**			**Strength of recommendation**,^**a**^ **median score (mean score)**	**Level of consensus**^**b**^
***CR14:*** *Attempts should be made to be fully inclusive in terms of diversity and disease-specific criteria. Underserved patients include those listed below, and barriers to their inclusion should be identified and removed:* • *Patients with low-risk disease* • *Patients with accelerated-phase myeloproliferative neoplasm (MPN)* • *Young and elderly patients* • *Patients with well-controlled human immunodeficiency virus (HIV)/viral hepatitis* • *Patients relapsing after allogeneic stem cell transplantation (allo-SCT)*			8 (8.08)	94.44% n/N = 36/38
**Q15. How can clinical trial inclusion criteria/endpoints be improved?**				
***CR15:*** *Efforts should focus upon validation of endpoints other than spleen volume and symptoms, such as, but not limited to:* • *Overall survival* • *Progression-free survival* • *Leukemia-free survival* • *Event-free survival* • *Time to treatment failure* • *Transfusion independence* • *Reduction of driver and/or additional mutation variant allele frequency* • *Improvement of bone marrow fibrosis grade* • *Normalization of cytokine levels* • *Artificial intelligence (AI)-based assessment of marrow morphology response* *Furthermore, given MF is a rare disease, the development of real-world evidence controls should be considered*.			8.5 (8.25)	95.83% n/N = 24/38

^a^Median score on a 1–9 scale (mean score in parentheses).

^b^Percentage of votes with 7–9 on a 9-point scale. Participants were provided with the voting option ‘Not Applicable’ for recommendations outside their area expertise; this option was selected by some patients.

^c^Since completion of the voting rounds, momelotinib was approved by the FDA and EMA for use in patients with MF and anemia, and can be used in patients with a platelet count of <50 × 10^9^/l.

Following the first voting round, consensus was achieved among voters (hematologists [*n* = 29] and patients [*n* = 9] from Europe, the United States, Canada, Australia, Israel, and India) for all 15 recommendations. Upon reviewing the feedback from voters who scored recommendations ≤6, and in the interest of producing the best outcome, the SC amended nine of the 15 recommendations for a second round of voting, after which consensus was achieved among voters (hematologists [*n* = 21] and patients [*n* = 5]) for eight of the nine recommendations that were submitted for revote. Following amendment of the remaining recommendation for a third voting round, consensus was achieved among voters (hematologists [*n* = 20] and patients [*n* = 4])—13 recommendations achieved consensus in the range of 90–100% and two in the range of 80–90%. The CRs are provided in Table 1, with a full description of feedback from each voting round in Supplementary Tables S2–S9. The recommendations are not intended to replace or modify/update existing guidelines.

Theme 1 (CR1–CR4) focused on optimizing anemia management in MF. Diagnosing and addressing anemia in patients with MF is complex, as it is frequently multifactorial and may be disease and/or treatment related [13]. A comprehensive workup, accurate diagnosis, and appropriate treatment are of critical importance. All current and emerging treatment options for MF- and treatment-related anemia should be considered and are outlined in Table 2. Importantly, only MMB is approved specifically for MF-related anemia [11, 12]. In addition to JAKi monotherapy, existing data support the efficacy of RUX-based combination therapies in managing MF with anemia; clinical trials featuring RUX-based combination therapies are summarized in reference [14].

**Table 2:** Current and emerging treatments for anemia in myelofibrosis.

Current treatments		
Treatment	Description	Examples
RBC transfusion	Replacement of or supplementation with mature RBCs by transfusion to increase Hb levels	
ESAs	Stimulate erythropoiesis by activating pro-erythroid signaling	Recombinant ESA
Androgens	Androgens have pro-erythroid properties that help mitigate MF-related anemia	Danazol
Splenectomy	Removal of the spleen to address splenomegaly and consequent destruction of mature RBCs	
IMiD^®^ agents	Immunomodulatory treatments with pleiotropic effects, including promoting erythropoiesis	Thalidomide Lenalidomide Pomalidomide
JAK inhibitors	Selective JAK inhibitors that prevent overactive JAK/STAT signaling due to mutation; though approved by the FDA as a treatment for MF that reduces splenomegaly, these can also exacerbate MF-related anemia	Ruxolitinib (JAK1/2 inhibitor) Fedratinib (JAK2 inhibitor) Pacritinib (JAK2, IRAK1 and ACVR1 inhibitor) Momelotinib (JAK1/2 and ACVR1 inhibitor)
**Emerging treatments**		
TGF-β ligand traps	Disrupt activated TGF-β/Smad signaling to prevent apoptosis of erythroblasts and promote erythroblast maturation	Luspatercept KER-050
Epigenetic modulators	BET inhibition can dampen inflammatory responses that disrupt the bone marrow microenvironment and contribute to anemia	Pelabresib
Antifibrotic agents	Addressing bone marrow fibrosis normalizes the bone marrow microenvironment to enhance effective erythropoiesis and prevent further expulsion of erythropoietic tissue	PRM-151
Telomerase inhibitors	Inhibition of telomerase in malignant HSPCs contributes to their selective elimination and allows for the normal production of RBCs	Imetelstat
BCL-2/BCL-xL inhibitors	Inhibition of the pro-apoptotic factors BCL-2/BCL-xL reduces apoptosis of maturing RBCs	Navitoclax

Adapted from Passamonti F, et al. Crit Rev Oncol Hematol. 2022; 180:103862 [13].

*ACVR1* activin A receptor type 1, *BET* bromodomain and extra-terminal domain, *ESA* erythropoiesis-stimulating agent, *FDA* US Food and Drug Administration, *Hb* hemoglobin, *HSPC* hematopoietic stem and progenitor cell, *IMiD* immunomodulatory drug, *IRAK1* interleukin-1 receptor-associated kinase 1, *JAK* Janus kinase, *MF* myelofibrosis, *RBC* red blood cell, *STAT* signal transducer and activator of transcription, *TGF-β* transforming growth factor β.

Theme 2 (CR5–CR7) considered the management of patients with MF and thrombocytopenia. Recommendations from the German Society of Hematology and Medical Oncology and the Society of Thrombosis and Hemostasis Research [15] can be used as a foundation for treatment decisions for patients with thrombocytopenia. MMB and PAC are the only JAKis specifically approved for use in patients with severe thrombocytopenia (at platelet counts ≥25 × 10^9^/l and <50 × 10^9^/l, respectively) [10–12]. For patients with platelet counts of 50–100 × 10^9^/l, the options are less clear. Consideration should be given to JAKi dose optimization/intensity, severity of anemia, tolerance of worsening thrombocytopenia, and concomitant medications, and be balanced against the reason for treatment.

Theme 3 (CR8–CR11) discussions included defining JAKi failure; while the stringent criteria for RUX failure (Supplementary Table S10) serve as a useful starting point, collaborative efforts should focus on their refinement to accurately differentiate intolerance and resistance, reflecting real-world clinical scenarios and a patient’s overall condition, and be applicable to JAKis other than RUX. Defining suboptimal response was challenging due to variable disease presentation and treatment goals, but was conceptualized as JAKi therapy benefiting the patient in certain aspects, but not meeting specified therapeutic targets. Parameters for assessing treatment response were then explored, as well as strategies for treatment switching. Importantly, physicians should not feel bound by definitions of relapsed, refractory, and suboptimal response; they are a guide to help inform the timing of potential treatment switches.

Theme 4 (CR12 and CR13) examined the utility of prognostic scoring tools (Supplementary Table S11) and clinical variables in predicting survival benefits, to guide clinical decision-making. Theme 5 (CR14 and CR15) emphasized the need for broader inclusion criteria in clinical trials, while advocating for improved trial endpoints to enhance their relevance and applicability in real-world clinical settings.

Patient insights should be central to shaping the MF diagnosis and treatment landscape, and patients in the EF involved in this program understood the importance of recommendations that help to ensure consistent and comprehensive care. Including patients in the EF ensured appropriate consideration was given to aspects outside a strictly medical viewpoint, such as burden of illness/therapy and quality of life. Patients highlighted the importance of effective communication and the development of trust between patients and healthcare professionals; this, in turn, facilitates patients’ active involvement in their own care, and effective shared decision-making based on mutual understanding. Patients want to know their condition is frequently and consistently monitored, and that their best interests remain the focal point of care.

The recommendations presented herein provide an up-to-date overview of contemporary MF treatment, offering a valuable supplement to existing treatment guidelines without aiming to replace them. A strength of this program lies in its recruitment of a diverse, international group, comprising an SC of nine expert hematologists and an EF of 20 hematologists and nine patients, thus ensuring robustness of the process and the incorporation of the patient perspective. The rigorous development process, involving multiple rounds of enhancements and revoting, highlighted the prevailing ambiguity and variability in current clinical practices, and underscored the importance of recommendations to guide and standardize care.

A limitation of these recommendations pertains to the relatively small number of experts and patients who actively participated in the voting process. Furthermore, an inherent weakness of the consensus-building process emerged, marked by a drop-off in participation during the voting rounds. However, this challenge is expected when accommodating diverse perspectives and expertise. Additionally, at present, there is a lack of available data for certain agents discussed herein in the context of MF. This gap indicates the need for continued research and data collection to inform comprehensive clinical decision-making.

Finally, since completion of the voting rounds, MMB was approved by the FDA and EMA as the first treatment for adults with intermediate- or high-risk MF and anemia. While clinical recommendations were initially crafted prior to its approval, our work was revised in acknowledgment of this approval, with all co-authors contributing to these revisions.

In this collaboration between an international panel of physicians with expertise in MF and a diverse EF, a high level of consensus was achieved on recommendations addressing a wide range of critical gaps in MF management. These recommendations provide a valuable framework to support clinicians in optimizing care for patients with MF.

### Additional information

The third party material in this article (Table 2 and Supplementary Table S10) is licensed under the Creative Commons Attribution 4.0 International License, which permits use, sharing, adaptation, distribution and reproduction in any medium or format, as long as you give appropriate credit to the original author(s) and the source, provide a link to the Creative Commons license, and indicate if changes were made. To view a copy of this license, visit https://creativecommons.org/licenses/by/4.0/.

### Supplementary information

Supplement

## Data Availability

The authors confirm that the data supporting the findings of this study are available within the article and/or its supplementary materials.
