# Supplementary material for: Myelofibrosis management in routine clinical practice with a focus on patients with cytopenias: recommendations from a global consensus group
Source: Leukemia. 2024 Jul 9;38(8):1831–8. doi: 10.1038/s41375-024-02330-7 (PMC11286526; doi:10.1038/s41375-024-02330-7)
Supplement: Supplementary file 1 — Supplement [file 41375_2024_2330_MOESM1_ESM.docx]

Supplementary Information

**23-LEU-1472RR**

**Myelofibrosis Management in Routine Clinical Practice With a Focus on Patients With Cytopenias: Recommendations From a Global Consensus Group**

*Harrison et al.*

Table of contents:

- Supplementary Table S1, pages 2–17
- Supplementary Tables S2–S9, pages 18–37
- Supplementary Table S10, page 38
- Supplementary Table S11, pages 39–41
- Supplementary References, page 42

**Supplementary Table S1. Further reading on the consensus themes**

| **Authors** | **Title** | **Publication and date** | **Article type** |
| --- | --- | --- | --- |
| *Defining the Thresholds for Anemia, and When to Initiate/Modify Treatment* | | | |
| [Al-Ali et al. 2020](https://pubmed.ncbi.nlm.nih.gov/32017044/) | Primary analysis of JUMP, a phase 3b, expanded-access study evaluating the safety and efficacy of ruxolitinib in patients with myelofibrosis, including those with low platelet counts | Br J Haematol 2020;189:888–903 | Clinical trial |
| [Cervantes et al. 2015](https://pubmed.ncbi.nlm.nih.gov/26122869/) | Danazol therapy for the anemia of myelofibrosis: Assessment of efficacy with current criteria of response and long-term results | Ann Hematol 2015;94:1791–6 | Clinical trial |
| [Gerds et al. 2020](https://www.ncbi.nlm.nih.gov/pmc/articles/PMC7686901/) | Determining the recommended dose of pacritinib: Results from the PAC203 dose-finding trial in advanced myelofibrosis | Blood Adv  2020;4:5825–35 | Randomized controlled trial |
| [Gupta et al. 2016](https://pubmed.ncbi.nlm.nih.gov/27587385/) | The impact of anemia on overall survival in patients with myelofibrosis treated with ruxolitinib in the COMFORT studies | Haematologica 2016;101:e482–4 | Randomized controlled trial |
| [Verstovsek  et al. 2023](https://pubmed.ncbi.nlm.nih.gov/36709073/) | Momelotinib versus danazol in symptomatic patients with anaemia and myelofibrosis (MOMENTUM): Results from an international, double-blind, randomised, controlled, phase 3 study | Lancet 2023;401:269–80 | Clinical trial |
| [Mascarenhas et al. 2018](https://www.ncbi.nlm.nih.gov/pmc/articles/PMC5885169/) | Pacritinib vs best available therapy, including ruxolitinib, in patients with myelofibrosis: A randomized clinical trial | JAMA Oncol 2018;4:652–9 | Randomized controlled trial |
| [Oh et al. 2022](https://ashpublications.org/blood/article/140/Supplement%201/1518/490663/Pacritinib-Is-a-Potent-ACVR1-Inhibitor-with) | Pacritinib is a potent ACVR1 inhibitor with significant anemia benefit in patients with myelofibrosis | Blood  2022;140:1518–21 | Post hoc analysis |
| [Mesa et al. 2017](https://www.ncbi.nlm.nih.gov/pmc/articles/PMC6553796/) | SIMPLIFY-1: A phase III randomized trial of momelotinib versus ruxolitinib in Janus kinase inhibitor-naïve patients with myelofibrosis | J Clin Oncol 2017;35:3844–50 | Clinical trial |
| [Stegelmann et al. 2022](https://www.ncbi.nlm.nih.gov/pmc/articles/PMC9429480/) | P1055: Clinical and genetic results of the phase Ib/II trial MPNSG-0212: Ruxolitinib plus pomalidomide in myelofibrosis with anemia | Hemasphere  2022;6:945–6 | Clinical trial |
| [Hernández-Boluda  et al. 2017](https://pubmed.ncbi.nlm.nih.gov/28009442/) | Predictive factors for anemia response to erythropoiesis-stimulating agents in myelofibrosis | Eur J Haematol 2017;98:407–14 | Clinical trial |
| [Cervantes et al. 2021](https://pubmed.ncbi.nlm.nih.gov/34017073/) | Efficacy and safety of a novel dosing strategy for ruxolitinib in the treatment of patients with myelofibrosis and anemia: The REALISE phase 2 study | Leukemia 2021;35:3455–65 | Clinical trial |
| [Gerds et al. 2022](https://ascopubs.org/doi/10.1200/JCO.2022.40.16_suppl.7061) | Thrombocytopenic myelofibrosis patients previously treated with a JAK inhibitor in a phase 3 randomized study of momelotinib versus danazol (MOMENTUM) | J Clin Oncol 2022;40:7061 | Meeting abstract |
| [Harrison et al. 2018](https://pubmed.ncbi.nlm.nih.gov/29275119/) | Momelotinib versus best available therapy in patients with myelofibrosis previously treated with ruxolitinib (SIMPLIFY 2): A randomised, open-label, phase 3 trial | Lancet Haematol 2018;5:e73–81 | Clinical trial |
| [Verstovsek 2023](https://link.springer.com/article/10.1007/s00277-023-05126-4) | How I manage anemia related to myelofibrosis and its treatment regimens | Ann Hematol 2023;102:689–98 | Review |
| [Crisà et al. 2018](https://pubmed.ncbi.nlm.nih.gov/29984826/) | The use of erythropoiesis-stimulating agents is safe and effective in the management of anaemia in myelofibrosis patients treated with ruxolitinib | Br J Haematol 2018;182:701–4 | Short report |
| [Passamonti et al. 2022](https://pubmed.ncbi.nlm.nih.gov/36332787/) | Anemia in myelofibrosis: Current and emerging treatment options | Crit Rev Oncol Hematol 2022;180:103862 | Review |
| [Kiladjian et al. 2021](https://www.researchgate.net/publication/354237812_MPN-106_Improved_Transfusion_Independence_Rates_for_Momelotinib_vs_Ruxolitinib_in_Anemic_JAKi-Naive_Myelofibrosis_Patients_are_Independent_of_Baseline_Platelet_or_Transfusion_Status) | MPN-106: Improved transfusion independence rates for momelotinib vs ruxolitinib in anemic JAKi-naïve myelofibrosis patients are independent of baseline platelet or transfusion status | Clin Lymphoma Myeloma Leuk 2021;21:S353–4 | Retrospective analysis |
| *Defining the Threshold for Thrombocytopenia and When to Initiate/Modify Treatment* | | | |
| [Appelmann et al. 2016](https://pubmed.ncbi.nlm.nih.gov/26916570/) | Diagnosis, prevention, and management of bleeding episodes in Philadelphia-negative myeloproliferative neoplasms: Recommendations by the Hemostasis Working Party of the German Society of Hematology and Medical Oncology (DGHO) and the Society of Thrombosis and Hemostasis Research (GTH) | Ann Hematol 2016;95:707–18 | Review |
| [Mascarenhas et al. 2018](https://pubmed.ncbi.nlm.nih.gov/29522138/) | Pacritinib vs best available therapy, including ruxolitinib, in patients with myelofibrosis: A randomized clinical trial | JAMA Oncol 2018;4:652–9 | Randomized controlled trial |
| [Mascarenhas et al. 2021](https://ashpublications.org/blood/article/138/Supplement%201/3639/479821/A-Retrospective-Head-to-Head-Comparison-between) | A retrospective head-to-head comparison between pacritinib and ruxolitinib in patients with myelofibrosis and moderate to severe thrombocytopenia | Blood  2021;138:(Suppl. 1) 3639 | Retrospective analysis |
| [Harrison et al. 2022 (P1069)](https://europepmc.org/article/PMC/PMC9430568#free-full-text) | P1069: Retrospective comparison of patient outcomes on pacritinib versus ruxolitinib in patients with myelofibrosis and thrombocytopenia | Hemasphere  2022;6(Suppl.): 959–60 | Retrospective analysis |
| [Pemmaraju et al. 2022](https://ascopubs.org/doi/10.1200/JCO.2022.40.16_suppl.7058) | Risk-adjusted safety analysis of pacritinib in patients with myelofibrosis | J Clin Oncol 2022;40:7058 | Meeting abstract |
| [Verstovsek et al. 2022](https://pubmed.ncbi.nlm.nih.gov/34551507/) | Retrospective analysis of pacritinib in patients with myelofibrosis and severe thrombocytopenia | Haematologica 2022;107:1599–607 | Retrospective analysis |
| [Begna et al. 2011](https://www.ncbi.nlm.nih.gov/pubmed/21052089) | A phase-2 trial of low-dose pomalidomide in myelofibrosis | Leukemia 2011;25:301–4 | Clinical trial |
| [Mei et al. 2022](https://journals.lww.com/hemasphere/fulltext/2022/06003/abstract_book_for_the_27th_congress_of_the.1.aspx)  (P1044) | P1044: Real-world ruxolitinib treatment pattern in myelofibrosis patients with thrombocytopenia | Hemasphere 2022;6:1796–7 | Real-world data analysis |
| [Venugopal et al. 2022](https://pubmed.ncbi.nlm.nih.gov/35622972/) | The odyssey of pacritinib in myelofibrosis | Blood Adv 2022;6:4905–13 | Review |
| [Gupta et al. 2021](https://pubmed.ncbi.nlm.nih.gov/33210570/) | Analysis of predictors of response to ruxolitinib in patients with myelofibrosis in the phase 3b expanded-access JUMP study | Leuk Lymphoma 2021;62:918–26 | Clinical trial |
| *Defining JAK Inhibitor Failure and What Would Warrant Switching Treatment* | | | |
| [Bose, Verstovsek. 2020](https://pubmed.ncbi.nlm.nih.gov/32903304/) | JAK inhibition for the treatment of myelofibrosis: Limitations and future perspectives | Hemasphere 2020;4:e424 | Review |
| [Harrison et al. 2020](https://pubmed.ncbi.nlm.nih.gov/32129512/) | Fedratinib in patients with myelofibrosis previously treated with ruxolitinib: An updated analysis of the JAKARTA2 study using stringent criteria for ruxolitinib failure | Am J Hematol 2020;95:594–603 | Clinical trial |
| [Yacoub et al. 2022](https://pubmed.ncbi.nlm.nih.gov/36163982/) | MPN-075 efficacy and safety of add-on parsaclisib to ruxolitinib therapy in myelofibrosis patients with low versus higher baseline platelet counts: A subgroup analysis of data from a phase 2 study | Clin Lymphoma Myeloma Leuk 2022;22(Suppl. 2):S324 | Clinical trial |
| [Yacoub et al. 2022](https://ashpublications.org/blood/article/140/Supplement%201/579/488441/Efficacy-and-Safety-of-Add-on-Parsaclisib-to) | Efficacy and safety of add-on parsaclisib to ruxolitinib therapy in myelofibrosis patients with suboptimal response to ruxolitinib: Final results from a phase 2 study | Blood  2022;140:(Suppl. 1)579–82 | Clinical trial |
| [Maffioli et al. 2022](https://www.ncbi.nlm.nih.gov/pmc/articles/PMC8941454/pdf/advancesADV2021006889.pdf) | A prognostic model to predict survival after 6 months of ruxolitinib in patients with myelofibrosis | Blood Adv 2022;6:1855–64 | Observational study |
| [Mascarenhas et al. 2022](https://pubmed.ncbi.nlm.nih.gov/36163980/) | MPN-036 treatment failure and transition to next-line therapy in myelofibrosis: A modified Delphi panel approach | Clin Lymphoma Myeloma Leuk 2022;22(Suppl. 2):S323 | Expert consensus |
| [Saleh, Ribrag. 2023](https://pubmed.ncbi.nlm.nih.gov/36939633/) | An evaluation of fedratinib for adult patients with newly diagnosed and previously treated myelofibrosis | Expert Rev Hematol 2023;16:227–36 | Expert opinion |
| [Patel et al. 2015](https://pubmed.ncbi.nlm.nih.gov/26124496/) | Correlation of mutation profile and response in patients with myelofibrosis treated with ruxolitinib | Blood  2015;126:790–7 | Clinical trial |
| [Oh et al. 2021](https://ashpublications.org/blood/article/138/Supplement%201/3638/479833/Baseline-Serum-Ferritin-Differentially-Predicts) | Baseline serum ferritin differentially predicts W24 transfusion independence response for momelotinib and ruxolitinib in patients with myelofibrosis | Blood  2021;138(Suppl. 1):3638 | Retrospective analysis |
| [Krauth et al. 2018](https://www.ncbi.nlm.nih.gov/pmc/articles/PMC6132876/) | Ruxolitinib therapy for myelofibrosis in Austria | Wien Klin Wochenschr 2018;130:495–504 | Expert consensus |
| [Cervantes et al. 2013](https://pubmed.ncbi.nlm.nih.gov/24174625/) | Three-year efficacy, safety, and survival findings from COMFORT-II, a phase 3 study comparing ruxolitinib with best available therapy for myelofibrosis | Blood 2013;122:4047–53 | Clinical trial |
| [Devos et al. 2022](https://www.tandfonline.com/doi/epdf/10.1080/16078454.2021.2009645?needAccess=true&role=button) | Updated recommendations on the use of ruxolitinib for the treatment of myelofibrosis | Hematology 2022;27:23–31 | Review |
| [Guglielmelli et al. 2022](https://pubmed.ncbi.nlm.nih.gov/34521299/) | Adherence to ruxolitinib, an oral JAK1/2 inhibitor, in patients with myelofibrosis: Interim analysis from an Italian, prospective cohort study (ROMEI) | Leuk Lymphoma 2022;63:189–98 | Observational study |
| [Gerds et al. 2022](https://jnccn.org/view/journals/jnccn/20/9/article-p1033.xml) | Myeloproliferative Neoplasms, Version 3.2022, NCCN Clinical Practice Guidelines in Oncology | J Natl Compr Canc Netw  2022;20:1033–62 | Guidelines |
| [Gerds et al. 2023](https://www.nccn.org/guidelines/recently-published-guidelines) | Myeloproliferative Neoplasms, Version 3.2023, NCCN Clinical Practice Guidelines in Oncology | National Comprehensive Cancer Network 2023 | Guidelines |
| [Harrison et al. 2022](https://pubmed.ncbi.nlm.nih.gov/35180010/) | Addition of navitoclax to ongoing ruxolitinib therapy for patients with myelofibrosis with progression or suboptimal response: Phase II safety and efficacy | J Clin Oncol 2022;40:1671–80 | Clinical trial |
| [Palandri et al. 2020](https://pubmed.ncbi.nlm.nih.gov/31860137/) | Life after ruxolitinib: Reasons for discontinuation, impact of disease phase, and outcomes in 218 patients with myelofibrosis | Cancer 2020;126:1243–52 | Observational study |
| [Copher et al. 2022](https://pubmed.ncbi.nlm.nih.gov/35274711/) | Treatment patterns, health care resource utilization, and cost in patients with myelofibrosis in the United States | Oncologist 2022;27:228–35 | Retrospective study |
| *How and When to Determine Prognosis in Patients with MF* | | | |
| [Cervantes et al. 2009](https://pubmed.ncbi.nlm.nih.gov/18988864/) | New prognostic scoring system for primary myelofibrosis based on a study of the International Working Group for Myelofibrosis Research and Treatment | Blood 2009;113:2895–901 | Multicenter study |
| [Guglielmelli et al. 2017](https://ashpublications.org/blood/article/129/24/3227/36288/Presentation-and-outcome-of-patients-with-2016-WHO) | Presentation and outcome of patients with 2016 WHO diagnosis of prefibrotic and overt primary myelofibrosis | Blood 2017;129:3227–36 | Real-world study |
| [Finazzi et al. 2018](https://www.ncbi.nlm.nih.gov/pmc/articles/PMC6221891/pdf/41408_2018_Article_142.pdf) | Prefibrotic myelofibrosis: Treatment algorithm 2018 | Blood Cancer J 2018;8:104 | Review |
| [Passamonti et al. 2010](https://pubmed.ncbi.nlm.nih.gov/20008785/) | A dynamic prognostic model to predict survival in primary myelofibrosis: A study by the IWG-MRT (International Working Group for Myeloproliferative Neoplasms Research and Treatment) | Blood 2010;115:1703–8 | Observational study |
| [Gangat et al. 2011](https://pubmed.ncbi.nlm.nih.gov/21149668/) | DIPSS plus: A refined Dynamic International Prognostic Scoring System for primary myelofibrosis that incorporates prognostic information from karyotype, platelet count, and transfusion status | J Clin Oncol 2011;29:392–7 | Original report |
| [Mosquera-Orgueira et al. 2022](https://pubmed.ncbi.nlm.nih.gov/36570691/) | Machine learning improves risk stratification in myelofibrosis: An analysis of the Spanish Registry of Myelofibrosis | Hemasphere 2022;7:e818 | Registry study |
| [Tefferi et al. 2018](https://pubmed.ncbi.nlm.nih.gov/29708808/) | MIPSS70+ version 2.0: Mutation and karyotype-enhanced international prognostic scoring system for primary myelofibrosis | J Clin Oncol 2018;36:1769–70 | Correspondence |
| [Tefferi et al. 2018](https://pubmed.ncbi.nlm.nih.gov/29654267/) | GIPSS: Genetically inspired prognostic scoring system for primary myelofibrosis | Leukemia 2018;32:1631–42 | Observational study |
| [Grinfeld et al. 2018](https://www.nejm.org/doi/full/10.1056/NEJMoa1716614) | Classification and personalized prognosis in myeloproliferative neoplasms | N Engl J Med 2018;379:1416–30 | Multicenter study |
| [Gagelmann et al. 2019](https://pubmed.ncbi.nlm.nih.gov/30760453/) | Comprehensive clinical-molecular transplant scoring system for myelofibrosis undergoing stem cell transplantation | Blood 2019;133:2233–42 | Multicenter study |
| [Passamonti et al. 2017](https://pubmed.ncbi.nlm.nih.gov/28561069/) | A clinical-molecular prognostic model to predict survival in patients with post polycythemia vera and post essential thrombocythemia myelofibrosis | Leukemia 2017;31:2726–31 | Observational study |
| [Duminuco et al. 2023](https://pubmed.ncbi.nlm.nih.gov/36983189/) | Myelofibrosis and survival prognostic models: A journey between past and future | J Clin Med 2023;12:2188 | Review |
| [Gangat et al. 2023](https://www.ncbi.nlm.nih.gov/pmc/articles/PMC9813003/) | Determinants of survival and retrospective comparisons of 183 clinical trial patients with myelofibrosis treated with momelotinib, ruxolitinib, fedratinib or BMS-911543 JAK2 inhibitor | Blood Cancer J 2023;13:3 | Retrospective study |
| [Ajufo et al. 2023](https://ascopubs.org/doi/abs/10.1200/JCO.2023.41.16_suppl.7018?af=R) | Spleen volume reduction predicts overall survival in myelofibrosis patients on pacritinib but not best available therapy: PERSIST-2 landmark OS analysis | J Clin Oncol 2023;41:7018 | Meeting abstract |
| [Newberry et al. 2017](https://pubmed.ncbi.nlm.nih.gov/28674026/) | Clonal evolution and outcomes in myelofibrosis after ruxolitinib discontinuation | Blood 2017;130:1125–31 | Retrospective study |
| [Auteri et al. 2021](https://ashpublications.org/blood/article/138/Supplement%201/3626/479755/Spleen-and-Liver-Fibrosis-Is-Associated-to) | Spleen and liver fibrosis is associated to treatment response and prognosis in Philadelphia-negative chronic myeloproliferative neoplasms | Blood  2021;138(Suppl. 1):3626 | Single-center study |
| [Breccia et al. 2019](https://pubmed.ncbi.nlm.nih.gov/30515542/) | Impact of comorbidities and body mass index in patients with myelofibrosis treated with ruxolitinib | Ann Hematol 2019;98:889–96 | Clinical trial |
| [Kröger et al. 2021](https://pubmed.ncbi.nlm.nih.gov/34023851/) | Impact of prior JAK-inhibitor therapy with ruxolitinib on outcome after allogeneic hematopoietic stem cell transplantation for myelofibrosis: A study of the CMWP of EBMT | Leukemia 2021;35:3551–60 | Retrospective study |
| [Menghrajani et al. 2019](https://www.ncbi.nlm.nih.gov/pmc/articles/PMC6426689/) | Predictive models for splenic response to JAK-inhibitor therapy in patients with myelofibrosis | Leuk Lymphoma 2019;60:1036–42 | Retrospective study |
| [Mesa et al. 2012](https://ashpublications.org/blood/article/120/21/1733/102746/Improvement-in-Weight-and-Total-Cholesterol-and) | Improvement in weight and total cholesterol and their association with survival in ruxolitinib-treated patients with myelofibrosis from COMFORT-I | Blood  2012;120:1733 | Post hoc analysis |
| [Mesa et al. 2021](https://ascopubs.org/doi/10.1200/JCO.2021.39.15_suppl.7046) | Association of transfusion independence with improved overall survival in myelofibrosis patients receiving momelotinib | J Clin Oncol 2021;39:7046 | Meeting abstract |
| [Miller et al. 2017](https://pubmed.ncbi.nlm.nih.gov/28606598/) | Practical measures of clinical benefit with ruxolitinib therapy: An exploratory analysis of COMFORT-I | Clin Lymphoma Myeloma Leuk 2017;17:479–87 | Clinical trial |
| [Palandri et al. 2020](https://pubmed.ncbi.nlm.nih.gov/32271957/) | Risk factors for progression to blast phase and outcome in 589 patients with myelofibrosis treated with ruxolitinib: Real-world data | Hematol Oncol 2020;38:372–80 | Observational study |
| [Verstovsek et al. 2022](https://ashpublications.org/blood/article/140/Supplement%201/6803/492685/Transfusion-Independence-Response-As-a-Potential) | Transfusion independence response as a potential surrogate for overall survival in JAKi-experienced patients with myelofibrosis from MOMENTUM | Blood 2022;140(Suppl. 1):6803–5 | Clinical trial |
| *Unmet Needs in MF Clinical Trials* | | | |
| [Gerds et al. 2022](https://pubmed.ncbi.nlm.nih.gov/35256316/) | Disease and clinical characteristics of patients with a clinical diagnosis of myelofibrosis enrolled in the MOST study | Clin Lymphoma Myeloma Leuk 2022;22:e532–40 | Observational study |
| [Palandri et al. 2021](https://www.ncbi.nlm.nih.gov/pmc/articles/PMC8189619/pdf/hs9-5-e566.pdf) | EP1092 impact of bone marrow fibrosis grade on  response and outcome in patients with primary  myelofibrosis treated with ruxolitinib: A post-hoc analysis of the JUMP study | Hemasphere 2021;5:522–3 | Post hoc analysis |
| [Bao et al. 2023](https://pubmed.ncbi.nlm.nih.gov/36812897/) | Patient-reported outcomes in young adults with myeloproliferative neoplasms | Acta Haematol 2023;146:293–306 | Multicenter study |
| [Pemmaraju et al. 2022](https://www.ncbi.nlm.nih.gov/pmc/articles/PMC9322520/pdf/CNCR-128-2420.pdf) | Defining disease modification in myelofibrosis in the era of targeted therapy | Cancer 2022;128:2420–32 | Review |
| [Ryou et al. 2023](https://www.nature.com/articles/s41375-022-01773-0) | Continuous indexing of fibrosis: Improving the assessment and classification of MPN patients | Leukemia 2023;37:348–58 | Computational study |

ACVR1, activin A receptor, type I; CMWP, Chronic Malignancies Working Party; DGHO, German Society of Hematology and Medical Oncology; DIPSS, Dynamic International Prognostic Scoring System; EBMT, European Society for Blood and Marrow Transplantation; GIPSS, Genetically Inspired Prognostic Scoring System; GTH, Society of Thrombosis and Hemostasis Research; IWG-MRT, International Working Group for Myeloproliferative Neoplasms Research and Treatment; JAK(i), Janus kinase (inhibitor); MF, myelofibrosis; MIPSS70+, Mutation and Karyotype-Enhanced International Prognostic Scoring System; MPN, myeloproliferative neoplasm; NCCN, National Comprehensive Cancer Network; OS, overall survival; WHO, World Health Organization.

**Voting Process and Extended Faculty Comments**

In the first round of voting by the Extended Faculty (EF), all recommendations reached consensus (75% of votes in the range 7–9 on a 9-point scale). However, valuable feedback was received from the EF, which the Steering Committee wished to use to further strengthen the recommendations; this led to the amendment of nine of the 15 recommendations and resubmitting them for a second round of voting. Additionally, CR13 underwent a second amendment, and was submitted for a third vote before finalization.

The comments provided by the EF in the first round (Table S2–S6) and second round of voting (S7–S9) are provided in the tables below. No additional comments were received in the second round of voting for CR15, nor in the third round of voting for CR13. The statements shown in the tables are the original versions commented upon, and so do not match the finalized recommendations.

**Supplementary Table S2. Voting Round 1: Defining the Thresholds for Anemia, and When to Initiate/Modify Treatment – verbatim reasons provided for scoring a clinical recommendation 6 or less**

| **Q1: What is the appropriate workup for anemia diagnosis in a patient with MF?** |
| --- |
| **CR1:** Anemia in MF is frequently multifactorial; workup should include evaluation of iron/vitamin B_12_/folate levels, exclusion of hemolysis and active bleeding, assessment for disease progression and any other comorbidity (see table below), and exclusion of treatment effect.  Summary of Diagnostic Tests for Anemia Workup in Patients with MF   \| **Primary diagnostic workup** \| **Diagnostic test** \| **Additional information** \| \| --- \| --- \| --- \| \| **Initial workup** \| **CBC, reticulocytes, differential (blood film)** \| - Mandatory to decide on second line of investigation, based on Hb, MCV, and RDW; review disease progression, eg blasts (if these are detected, repeat karyotype, genetics, and BM biopsy may be needed) \| \| **EPO level** \|  \| \| **Iron/vitamin B_12_/folate levels** \|  \| \| **Renal profile** \|  \| \| **Exclude active bleeding** \| **Stool and urine testing, imaging, or endoscopy** \|  \| \| **Exclude hemolytic anemia** \| **Peripheral blood film and reticulocyte count** \| - Abnormalities of RBC morphology often suggest the presence of hemolysis, but are difficult to evaluate with concurrent MF - Exclude PNH \| \| **Serum bilirubin, haptoglobin, and ALT levels** \| - Elevated indirect bilirubin with normal ALT \| \| **Coombs test** \| - Both direct and indirect Coombs testing should be performed, including testing for the presence of C3d. If the patient has received a transfusion in the last 3 months, a positive result could also indicate alloantibodies to transfused RBCs (usually occurring in an acute or delayed hemolytic reaction) \| \| **Exclude hemoglobinopathies** \| **Hb electrophoresis** \| - To be performed if clinically indicated \|   *ALT, alanine transaminase; BM, bone marrow; C3, complement component 3; CBC, complete blood count;  EPO, erythropoietin; Hb, hemoglobin; LDH, lactate dehydrogenase; MCV, mean corpuscular volume; PNH, paroxysmal nocturnal hemoglobinuria; RBC, red blood cell; RDW, red cell distribution width.* |
| **Reasons provided for scoring the recommendation 6 or less:**  *“No need to evaluate for hemoglobinopathies or hemolytic anemia unless clinically suspected”*  *Such an extensive evaluation is not necessary in every MF patient with anemia. For example, C3b testing? Excluding PNH? Hb electrophoresis? Those tests are really not applicable to most patients”*  *“Would only recommend Coombs test if evidence of hemolysis”*  *“EPO levels rarely useful unless impaired renal function and not available/funded in many countries unless there is significantly low creatinine clearance. I would only do them in patients with creatinine clearance <50. Folate deficiency is very rare – our lab doesn't test for it unless clear indication, eg active prolonged haemolysis”*  *“No need for assessment of bleeding, eg with endoscopy if iron levels normal. Coombs test, haptoglobin, etc. irrelevant if no evidence of haemolysis on reticulocyte count. Hence investigation needs to be individualised”* |
| **Q2. When should treatment (that is not transfusion based) be initiated/modified to improve anemia? Which patient characteristics should be considered?** |
| **CR2:** Treatment for anemia (that is not transfusion based) should be considered for patients with a hemoglobin (Hb) level of ≤10g/dl, and in some cases at higher Hb levels; for example, anemia following initiation of therapy (eg Janus kinase [JAK] inhibitor) should be anticipated and therapy dose should be optimized. For persistent anemia after JAK inhibitor dose optimization, consider addition of a treatment, such as erythropoietin-stimulating agents (ESAs) for patients with EPO levels <500 IU, or treatment with an agent such as danazol that abrogates anemia, if EPO levels are >500 IU or the patient is refractory to ESAs. At present, there are no approved treatments for  MF-associated anemia. |
| **Reasons provided for scoring the recommendation 6 or less:**  *“Luspatercept is also an option for treating MF-associated anemia. ESAs and danazol are both mentioned as off-label options”*  *“Symptoms or comorbidities relevant to anemia should be considered rather than a single threshold”*  *“The evidence for EPO benefit is predominantly in patients with levels <125 IU/l. Treatment for anaemia should be based on symptoms rather than a particular level. Danazol may not be an optimal drug in women. Note that the anaemia secondary to ruxolitinib often improves with time so not necessarily an indication to intervene early. It’s not clear what is meant by optimisation of the ruxolitinib dose for anaemia... I'm not aware of any specific guidelines, unlike for thrombocytopenia”*  *“The decision to treat anemia is a clinical one. Not all patients require anemia treatment”* |
| **Q3: Which current and emerging treatments to improve anemia should be considered for:**   - **MF-related anemia** - **Treatment-related anemia** |
| **CR3:** Once other causes such as disease progression have been excluded: for MF-related anemia, JAK inhibition with momelotinib or pacritinib, danazol, ESAs, immunomodulatory imide drugs (IMiDs), or conventional combination therapies may overcome the necessity of dose adjustments/interruptions, which may be associated with ruxolitinib or fedratinib. In the future, novel combination therapies may deliver these benefits. Splenectomy can be considered as a last resort in extreme cases of refractory disease-related anemia. For treatment-related anemia consider dose reduction of current therapy for 4–6 weeks. |
| **Reasons provided for scoring the recommendation 6 or less:**  *“Pacritinib and momelotinib currently not approved in EU”*  *“Splenectomy will only help in cases where there is hemolysis. Combining IMiDs with JAK inhibitors is not advised as it leads to excess toxicity. Lastly, would also mention luspatercept as it can be combined with JAK [inhibitors] and responses have been observed in patients who have had prior ESA/danazol”*  *“Splenectomy is not a measure of last resort for anemia as it won’t improve counts and can often times worsen counts. In addition, it is associated with significant morbidity and mortality. Momelotinib is not approved yet, thus can't be part of a consensus statement. Not clear what is meant by conventional combination therapies”*  *“Drugs such as momelotinib, ESAs, IMiDs are not readily available in many countries… These guidelines need to acknowledge that there may be one set of guidelines for the US and another for elsewhere”*  *“I would only dose reduce RUX if the anaemia was symptomatic, as outlined above... Arguably could continue presumed therapeutic dose and give a transfusion until the anaemia improves with time”*  *“Splenectomy is very controversial… …need to define ‘extreme case’, eg massive splenomegaly with minimally durable response to transfusions in a patient surgically fit”*  *“Low-quality evidence for IMiDs, except perhaps in del(5q). MMB or PAC not clearly superior to RUX/FED overall, so need to weigh up global impact of TKI effects on SVR/TSS vs anaemia when deciding on therapy, whereas this statement seems to prioritise anaemia impact over other aspects of disease”*  *“After 4–6 weeks at reduced dose… shall we recommend to increase the dose even if anemia does not improve?”* |
| **Q4. Aside from access and reimbursement, what factors guide selection of JAK inhibitor therapy in patients with MF and anemia?** |
| **CR4:** Factors guiding selection of JAK inhibitor monotherapy would include:   - Baseline Hb - Likely tolerance of anemia - Baseline thrombocytopenia - Spleen size and symptoms   For some agents, consideration of drug-specific adverse events (immunosuppression, skin cancer, infection risk, nutritional status, tolerance of gastrointestinal [GI] toxicity, neurotoxicity, cardiovascular adverse events) is also a factor. These factors are relevant for first- and second-line therapy. |
| **Reasons provided for scoring the recommendation 6 or less:**  *“Agree with 4 main points, but unclear whether we have sufficient evidence to say that immunosuppression, skin cancer, perhaps some other toxicities really are drug specific. Others (GI toxicity, neuropathy) more clearly are drug specific”* |

ALT, alanine transaminase; BM, bone marrow; C3, complement component 3; CBC, complete blood count; EPO, erythropoietin; ESA, erythropoietin-stimulating agent; EU, European Union; FED, fedratinib; GI, gastrointestinal; Hb, hemoglobin; IMiD, immunomodulatory imide drug; JAK, Janus kinase; MCV, mean corpuscular volume; MF, myelofibrosis; MMB, momelotinib; PAC, pacritinib; PNH, paroxysmal nocturnal hemoglobinuria; RBC, red blood cell; RDW, red cell distribution width; RUX, ruxolitinib; SVR, spleen volume reduction; TKI, tyrosine kinase inhibitor; TSS, total symptom score.

**Supplementary Table S3. Voting Round 1: Defining the Threshold for Thrombocytopenia and When to Initiate/Modify Treatment – verbatim reasons provided for scoring 6 or less on a clinical recommendation**

| **Q5: Which treatments for MF can be safely administered to patients with thrombocytopenia, and when should treatment be initiated/modified?** |
| --- |
| **CR5:** Management of spleen, symptoms, and anemia in patients with MF and a platelet count of <50×10^9^/l is complex. There are reports that the use of pacritinib, momelotinib, and ruxolitinib may be feasible in this setting. It is important to consider the risk of bleeding associated with thrombocytopenia and concomitant use of anticoagulation/antiplatelet therapy, and, possibly, consider prophylaxis with antifibrinolytics. |
| **Reasons provided for scoring the recommendation 6 or less:**  *No rationale to justify the use of prophylaxis with antifibrinolytics in this setting. Furthermore, ruxolitinib – which should be administered at low doses in the cytopenic patient – should not be listed as one of the possible choices on a par with other JAK inhibitors”*  *“Pacritinib and momelotinib are currently not approved in the EU”*  *‘Important to consider the risk of bleeding ... antiplatelet therapy’ statement doesn't need to be said here as a standalone observation. It could be added to the end of the first sentence ‘particularly in patients on concomitant anticoagulation/antiplatelet therapy’. The pacritinib approval includes patients with counts <50; ruxolitinib does not. Important to consider other causes of thrombocytopenia, eg liver disease from hepatic or portal vein thrombosis with low TPO production”*  *“Pacritinib is approved in this setting so saying that there are ‘reports’ to support its use is inaccurate”*  *“There are RCTs for PAC and MMB in this population and studies (early phase) for ruxolitinib”*  *“Maybe we should not mention prophylaxis with antifibrinolytics since there isn’t supporting data and it may have potential adverse events”*  *“Would modify to low-dose ruxolitinib”*  *“1) It may not be appropriate here to recommend use of ruxolitinib for patients with PLT<50” 2) FED can be used for patients with PLT counts >25, I would include this info”* |
| **Q6. Which treatments to increase platelet count can be safely administered to patients with MF and thrombocytopenia, and when should treatment be initiated/modified?** |
| **CR6:** Treatments to increase platelet counts are rarely effective; agents such as low-dose corticosteroids, danazol, or low-dose thalidomide could be considered. There are no data supporting the safety or benefit of thrombopoietin (TPO) mimetics in this setting. Splenectomy can be considered as a last resort in extreme cases. |
| **Reasons provided for scoring the recommendation 6 or less:**  *“Overall agree with the statement. Regarding the statement – no data supporting the safety or benefit of thrombopoietin: note that there is a small n=6 study of eltrombopag in MF, which showed that eltrombopag doesn’t work in this setting”*  *“Splenectomy should be used with caution as it is associated with significant morbidity and mortality, which is likely higher in a severely thrombocytopenic patient. Danazol will not improve thrombocytopenia”* |
| **Q7. Aside from access and reimbursement, what factors guide selection of JAK inhibitor therapy in patients with MF and thrombocytopenia?** |
| **CR7:** Factors guiding selection of JAK inhibitor monotherapy would include:   - Baseline Hb - Degree of thrombocytopenia (eg the only currently approved therapy for patients with platelets <50×10^9^/l is pacritinib) - Spleen size   Consideration should be given to dose optimization/intensity, severity of anemia, tolerance of worsening thrombocytopenia, and concomitant medication, and be balanced against the reason for treatment. For some agents, baseline nutritional status and tolerance of GI toxicity are also factors and require an effective mitigation plan. These factors are relevant for first- and second-line therapy. |
| **Reasons provided for scoring the recommendation 6 or less:**  *“What about the consideration of previous medication? Would it be possible to take symptoms into account?”*  *“I would add the assessment of renal function”*  *“Spleen size is not a factor in JAKi selection* per se” |

EU, European Union; FED, fedratinib; GI, gastrointestinal; Hb, hemoglobin; JAK(i), Janus kinase inhibitor; MF, myelofibrosis; MMB, momelotinib; PAC, pacritinib; PLT, platelet; RCT, randomized controlled trial; TPO, thrombopoietin.

**Supplementary Table S4. Voting Round 1: Defining JAK Inhibitor Failure and What Would Warrant Switching Treatment – verbatim reasons provided for scoring 6 or less on a clinical recommendation**

| **Q8: What criteria should be used to define a patient who is relapsed, refractory, or intolerant to JAK inhibitor treatment?** |
| --- |
| **CR8:** There are existing criteria for ruxolitinib that are used in clinical trials to determine if a patient is relapsed, refractory, or intolerant to treatment (see table below); however, in clinical practice, it may be difficult to distinguish exactly between ruxolitinib intolerance and relapse, as often these can go hand in hand. Criteria for other JAK inhibitors are likely to be similar. Criteria for ruxolitinib failure used in the re-analysis of the JAKARTA-2, PAC203, and FREEDOM trials  **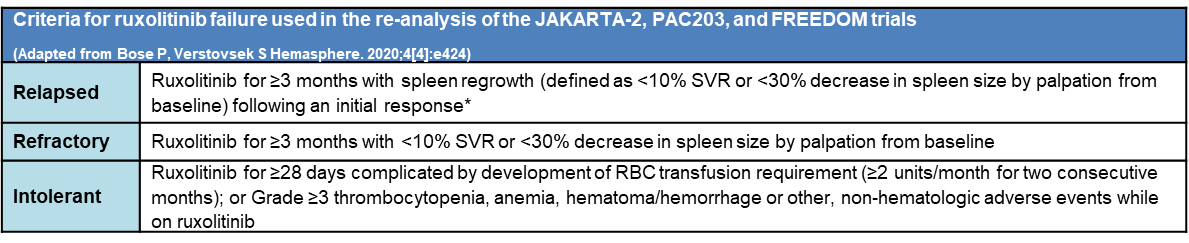** |
| **Reasons provided for scoring the recommendation 6 or less:**  *“Emphasis on SVR at the expense of symptoms/QoL/cytopenia improvement, which may be primary or co-objectives for some patients. Palpable spleen size criteria are problematic, and outside trials the reporting of volume is inconsistent in local practice. The SVR criteria are more reliable. Should there be a recommendation that volumetric reporting be routine for MF patients? Incorporating this in consensus recommendations may be useful to help bring this into routine radiology practice”* |
| **Q9. How is a suboptimal JAK inhibitor response defined?** |
| **CR9 version 1:** Suboptimal response to a JAK inhibitor could be defined as instances where the JAK inhibitor retains benefit in some aspects of disease but not others. For example, residual splenomegaly or symptoms, or failure to achieve an anemia response, where this is the target of therapy with drugs such as momelotinib or pacritinib. |
| **Reasons provided for scoring the recommendation 6 or less:**  *“The second version is better based on the specificity”*  *“Pacritinib and momelotinib are currently not approved in the EU”*  *“Spleen size at start of therapy varies widely between patients. A dramatic reduction in size does not necessarily mean it becomes impalpable”*  *“Anemia is on target toxicity for ruxolitinib and fedratinib, and cannot be used as criteria for suboptimal response”*  *“Too generic”* |
| **CR9 version 2:** Suboptimal response to a JAK inhibitor can be defined using the criteria for ruxolitinib suboptimal response used in the phase II INCB50465-201 trial: Palpable spleen >10cm below left subcostal margin (LSM), or palpable spleen 5–10cm below LSM and active symptoms, after ≥6 months of receiving ruxolitinib (5–25mg twice daily [BID]; stable dose ≥8 weeks).  (Yacoub A, et al. Clin Lymphoma Myeloma Leuk. 2022; 22[Suppl. 2]:S324, Yacoub A, et al. Blood. 2022; 140[Suppl. 1]:579–82) |
| **Reasons provided for scoring the recommendation 6 or less:**  *“Too much focus on spleen size. A patient with only mild splenomegaly but severe symptoms, who has insufficient symptom response to ruxolitinib, should also be considered suboptimal”*  *“Total focus on spleen size ignores symptomatic benefits that patients often report”*  *“The first statement is more useful in clinical practice. Criteria of the second statement are more appropriate for clinical trials because it’s more restrictive and don’t address all relevant symptoms that can be improved on treatment”*  *“Spleen size at commencement of a JAK inhibitor varies widely between patients and using precise criteria as above is not optimal”*  *“Spleen palpation in unreliable”*  *“I think there are patients who could benefit from change that fall through these criteria and would favor a more all-encompassing set of criteria”*  *“Palpable spleen length unreliable assessment of disease burden”*  *“We are patients and we look more for symptoms than clinical values”* |
| **Q10. Which MF parameters should be incorporated into response assessments?  When should they be repeated and how often?** |
| **CR10:** Response assessments should be repeated every 3–6 months, depending on MF risk category and stability, and include:   - Accurately determined spleen size (eg using a measuring tape) - Symptom score with a validated tool such as the Myeloproliferative Neoplasm Symptom Assessment Form Total Symptom Score (MPN-SAF TSS) or the Myelofibrosis Symptom Assessment Form (MFSAF) - Full blood count, including blast percentage - Control of elevated blood counts - Degree of anemia and/or thrombocytopenia - Formalized disease prognostic risk assessment in patients of transplant-eligible age   There are no data for how often next-generation sequencing (NGS) or karyotyping should be performed, but alongside a repeated bone marrow biopsy this should be considered if disease progression is suspected upon discontinuation of therapy or changing line of therapy.  Other biomarkers such as cytokine levels, changes in fibrosis grade, or variant allele burden assessment are investigational. Monitoring of driver mutation variant allele frequency (VAF) is not currently recommended but may be important in the future. |
| **Reasons provided for scoring the recommendation 6 or less:**  *“Time is limited. I just talk to the patient in gen*e*ral. I'm not convinced that using a formal score adds any benefit in my assessment of disease status and management... Has use of these scores been proven to benefit outcome?”*  *“I also measure LDH and get a blood film to assess the degree of tear drop poikilocytosis. In patients on interferon with JAK2-positive disease I do use VAF to get some idea of response... but agree that there is little current evidence to support this as a reliable marker of disease response in MF”*  *“I understand from worldwide allogeneic SCT professionals that usually after several of 3–4 weeks from starting the full dose of JAK inhibitors, the effect on the spleen size is felt and measured and if it doesn’t work, it won't do so in the rest of the [3 months period]”*  *“I would eliminate formalized disease prognostic risk assessment in patients of transplant-eligible age”*  *“During JAKi therapy there is a fluctuation of blood counts and transient disappearance of constitutional symptoms. These treatment-related fluctuations may significantly alter (improve or worsen) available MF prognostic score models. Therefore, prognosis should be evaluated before JAKi therapy start”*  *“Spleen imaging should be recommended”* |
| **Q11. Which clinical characteristics determine when a treatment switch is warranted?** |
| **CR11:** Treatment should be given at a maximum tolerated dose for 3–6 months to determine best response. A treatment switch is warranted/should be considered in accordance with previous definitions for relapsed, refractory, suboptimal response, or a lack of maintained response. For intolerance or toxicity it should be noted that criteria may be reached sooner. Treating physicians should be cautious of discontinuation syndrome (best described with ruxolitinib). |
| **Reasons provided for scoring the recommendation 6 or less:**   - “The MTD concept seems a bit outdated and ill-defined” |

EU, European Union; JAK(i), Janus kinase (inhibitor); LDH, lactate dehydrogenase; MF, myelofibrosis; MFSAF, Myelofibrosis Symptom Assessment Form; MPN-SAF TSS, Myeloproliferative Neoplasm Symptom Assessment Form Total Symptom Score; MTD, maximum tolerated dose;
NGS, next-generation sequencing; QoL, quality of life; SCT, stem cell transplantation;
RBC, red blood cell; SVR, spleen volume reduction; VAF, variant allele frequency.

**Supplementary Table S5. Voting Round 1: How and When to Determine Prognosis in Patients With MF – verbatim reasons provided for scoring 6 or less on a clinical recommendation**

| **Q12: Which prognostic scoring tools should be used:**   - **For patients with pre-PMF** - **At diagnosis** - **During the course of MF disease** - **To determine transplantation risk** |
| --- |
| **CR12:** The table below contains prognostic scores for overt primary MF (PMF) or post-polycythemia vera/-essential thrombocytopenia (PV/-ET) MF that have been validated for use at diagnosis, during the course of disease, and to determine transplantation risk. It is important that clinical staff are aware of the limitations and appropriate use of these scores. The development of prognostic scores for patients with pre-PMF is an unmet need, although the International Prognostic Scoring System (IPSS) has been validated in this setting. Please see Table 4 in the main manuscript for ‘Summary of Prognostication Models Validated in Patients With Myelofibrosis’ |
| **Reasons provided for scoring the recommendation 6 or less:**  *“This is not a recommendation, but a collection of scores”* |
| **Q13. Which clinical variables are predictive of long-term outcome or survival benefit  in patients receiving JAK inhibitor therapy?** |
| **CR13:** The following variables may be useful in predicting the outcome for patients receiving JAK inhibitor therapy; however, it is important to consider the limitations of survival data:   - Spleen size reduction (for ruxolitinib and pacritinib) - Weight gain (for ruxolitinib) - Mutational complexity (for ruxolitinib) - Transfusion independence (for momelotinib) - Full blood count (Hb, platelet count, blasts) - Clonal progression (following discontinuation of ruxolitinib) |
| **Reasons provided for scoring the recommendation 6 or less:**  *“The content adheres to the literature, but I think the statement could be reworded more clearly. Some of the listed factors influence positively, others negatively. I would prefer to put them in a table that can help (also visually) in creating associations between parameter, reference drug and prognostic value”*  *“I would definitely add also:*   - *Age* - *Liver size (as it is also a dynamic factor after spleen size)* - *Other critical symptoms: night-sweats and bone-aches, and maybe (not sure if this secondary decrease is common in other MF patients) the prediction on the secondary acquired von Willebrand disease (AvWD)”*   *“Data for weight gain is highly confounded. Most patients outside are not cachexic and gain weight nonetheless. It is not known how ruxolitinib-induced weight gain affects survival in most patients”*  *“Spleen size reduction is observed in responding patients by definition. Lack of response being associated with shortened survival during ineffective (by definition) treatment is somewhat  self-referential”*  *“Momelotinib and pacritinib are currently not approved in the EU”*  *“I agree with the concept of the statement, but the way it is presented isn’t as clear. Maybe indicate what factors are associated with improved survival with ruxolitinib and momelotinib, but clarification of mutational complexity, full blood count and clonal progression is needed”* |

AvWD, acquired von Willebrand disease; ET, essential thrombocytopenia; EU, European Union;
Hb, hemoglobin; IPSS, International Prognostic Scoring System; JAK, Janus kinase;
MF, myelofibrosis; PMF, primary myelofibrosis; PV, polycythemia vera.

**Supplementary Table S6. Voting Round 1: Unmet Needs in MF Clinical Trials – verbatim reasons provided for scoring 6 or less on a clinical recommendation**

| **Q14: How can broader inclusion in clinical trials be achieved?** |
| --- |
| **CR14:** Attempts should be made to be fully inclusive in terms of diversity and disease-specific criteria. Underserved patients include those listed below and barriers to their inclusion should be identified and removed:   - Patients with low-risk disease - Patients with accelerated phase MPN - Young and elderly patients - Patients with well-controlled human immunodeficiency virus (HIV)/hepatitis - Patients relapsing after allogeneic stem cell transplantation (allo-SCT) |
| **Reasons provided for scoring the recommendation 6 or less:**  *“I'd also add:*   - *Patients who aren’t interested to do additional bone marrow biopsies (some are facing severe difficulties and bleeding after every additional biopsy)* - *Patients who aren’t interested to be CT/radiated scanned on routine basis. We've realized there is a huge difference in the numeric results of spleen US scan size vs CT scan. (Also for young women, the timing of the scan from the feminine period is also a factor that changes the accuracy)”*   *“Assuming that the optimal baseline of measuring spleen (and liver) size is by MRI scan (preferably without contrast material). In Israel it is still quite a challenge to confirm MRI spleen scan”*  *“How about under-represented minority groups?”* |
| **Q15. How can clinical trial inclusion criteria/endpoints be improved?** |
| **CR15:** Efforts should focus upon validation of additional endpoints such as, but not limited to:   - Transfusion independence - Reduction of driver and/or additional mutation variant allele frequency - Improvement of bone marrow fibrosis grade - Normalization of cytokine levels - Artificial intelligence (AI)-based assessment of marrow morphology response   Furthermore, given MF is a rare disease, the development of real-world evidence controls should be considered. |
| **Reasons provided for scoring the recommendation 6 or less:**  *“This isn’t as clear, although I like the idea of what it is trying to say: that these factors may be response criteria that are valid, but need further investigation”*  *“OS, PFS, leukemia-free survival should also be included”*  *“Overall survival, event-free survival, time to treatment failure must be endpoints until we have validated surrogates for these measures”*  *“**Transfusion independence (TI) is an important endpoint for those patients that are transfusion dependent”*  *“None of the measures mentioned (save TI) are validated. They are certainly among the more interesting exploratory measures (by no means all nor necessarily the best) but are reasonable. But the effort must be to develop surrogates WITH validation as surrogates for survival (as above). Without this we will continue to spin our wheels, spend money and waste time”*  *“Add instruments for biometric measurement of vital, anthropometric, and possibly biochemical parameters at home. Aso include translational research sub-projects for the identification of new markers related to disease modification. Other possible endpoints are the control of cytopenias, transformation rate, vascular events (both thrombotic and haemorrhagic) and OS prolongation”* |

AI, artificial intelligence; allo-SCT, allogeneic stem cell transplantation; CT, computed tomography; HIV, human immunodeficiency virus; MF, myelofibrosis; MPN, myeloproliferative neoplasm;
MRI, magnetic resonance imaging; OS, overall survival; PFS, progression-free survival;
US, ultrasound.

**Supplementary Table S7. Voting Round 2: Defining the Thresholds for Anemia, and When
to Initiate/Modify Treatment – verbatim reasons provided for scoring 6 or less on a clinical recommendation**

| **Q1: What is the appropriate workup for anemia diagnosis in a patient with MF?** |
| --- |
| **CR1:** Anemia in MF is frequently multifactorial; workup should include evaluation of iron/vitamin B_12_/folate levels, exclusion of hemolysis and active bleeding, assessment for disease progression and any other comorbidity (see table below), and exclusion of treatment effect. In cases of hemolysis, additional investigations are recommended. Please see Table 1 in the main manuscript for ‘Summary of Diagnostic Tests for Anemia Workup in Patients with MF’ |
| **Reasons provided for scoring the recommendation 6 or less:**  *“It is unclear whether this refers to newly diagnosed or progressive anaemia in a patient with pre-existing MF. This should be made clear. It also should be clear whether this is an isolated case of anaemia, ie not in the context of cytopenia in other lineages. Iron assessments should be part of this as bleeding is more common than haemolysis, for example. PNH must be incredibly rare and should only be considered if Coombs-negative active haemolysis. Gynaecological examination again is largely irrelevant... but may be required if significant menorrhagia is observed. Hb EP irrelevant as a haemoglobinopathy is very unlikely to appear in someone without pre-existing haemoglobinopathy recognised earlier. The criteria for a repeat marrow (assuming this is in a patient with pre-existing MF) should be discussed ... 'review disease progression, eg blasts (if these are detected, repeat karyotype, genetics, and BM biopsy may be needed)' is inadequate.. Could repeat marrow with karyotype, etc. should be performed if any of: progressive cytopenia in other lineages, increasing PB blasts, progressive splenomegaly and reticulocytopenia (PRCA has been reported, albeit rarely) in MF”* |
| **Q2. When should treatment (that is not transfusion based) be initiated/modified to improve anemia? Which patient characteristics should be considered?** |
| **CR2:** Treatment for anemia (that is not transfusion based) should be considered for patients with a hemoglobin (Hb) level of <10g/dl, and in some cases at higher Hb levels; for example, anemia following initiation of therapy (eg Janus kinase [JAK] inhibitor) should be anticipated and therapy dose should be optimized. For persistent anemia, consider addition of a treatment, such as erythropoietin-stimulating agents (ESAs) for patients with erythropoietin (EPO) levels <500 IU, or treatment with an agent such as danazol or luspatercept that abrogates anemia, if EPO levels are >500 IU or the patient is refractory to ESAs.  Use of these agents is currently off-label in this setting as, at present, there are no treatments specifically approved for MF-related anemia. |
| **Reasons provided for scoring the recommendation 6 or less:**  *“I wouldn't treat an asymptomatic Hb of 95g/l... Do not specify a level, but rather say 'symptomatic anaemia'. The anaemia after a JAK2 inhibitor is often reversible and not an indication to optimise the dose unless symptomatic”*  *“Should read ‘erythropoiesis-stimulating agents’ and not erythropoietin”* |
| **Q3: Which current and emerging treatments to improve anemia should be considered for:**   - **MF-related anemia** - **Treatment-related anemia** |
| **CR3:** Once other causes such as disease progression have been excluded: for MF-related anemia, Janus kinase (JAK) inhibition with momelotinib or pacritinib, danazol, luspatercept, erythropoietin-stimulating agents (ESAs), immunomodulatory drugs (IMiD^®^), or conventional combination therapies, such as JAK inhibition plus ESAs, danazol, luspatercept, or IMiD^®^, may overcome the necessity of dose adjustments/interruptions, which may be associated with ruxolitinib or fedratinib. In the future, novel combination therapies may deliver these benefits. Splenectomy can be considered as a last resort in select cases of refractory disease-related anemia. For treatment-related anemia consider dose reduction of current therapy for 4–6 weeks. |
| **Reasons provided for scoring the recommendation 6 or less:**  *Recommendation achieved 100% consensus* |
| **Q4. Aside from access and reimbursement, what factors guide selection of JAK inhibitor therapy in patients with MF and anemia?** |
| **CR4:** Factors guiding selection of Janus kinase (JAK) inhibitor monotherapy would include:   - Baseline hemoglobin (Hb) - Likely tolerance of anemia - Baseline thrombocytopenia - MF-related symptoms   For some agents, consideration of drug-specific adverse events (immunosuppression, skin cancer, infection risk, nutritional status, tolerance of gastrointestinal [GI] toxicity, neurotoxicity, cardiovascular adverse events) is also a factor. |
| ***Reasons provided for scoring the recommendation 6 or less:***  *“Drug-specific adverse events should be considered for ALL agents as a main factor to guide selection of JAK inhibitors”*  *“The 4 main factors are OK. The second statement implies that certain JAK inhibitors are specifically associated with certain events. Some of these are uncontroversial (eg neuropathy), but I am not convinced that there is a clearly defined differential risk of, eg infection, skin cancer, vascular risk”* |

BM, bone marrow; EP, erythropoiesis; EPO, erythropoietin; ESA, erythropoietin-stimulating agent;
GI, gastrointestinal; Hb, hemoglobin; IMiD, immunomodulatory imide drug; JAK, Janus kinase; MF, myelofibrosis; PB, peripheral blood; PNH, paroxysmal nocturnal hemoglobinuria; PRCA, pure red cell aplasia.

**Supplementary Table S8. Voting Round 2: Defining the Threshold for Thrombocytopenia and When to Initiate/Modify Treatment – verbatim reasons provided for scoring 6 or less on a clinical recommendation**

| **Q5: Which treatments for MF can be safely administered to patients with thrombocytopenia, and when should treatment be initiated/modified?** |
| --- |
| **CR5:** Management of spleen, symptoms, and anemia in patients with MF and a platelet count of 50–100×10^9^/l with pacritinib, fedratinib, momelotinib, or low-dose ruxolitinib is feasible. Management in patients with MF and a platelet count of <50×10^9^/l is complex. Pacritinib is approved by the US Food and Drug Administration for patients with a platelet count of <50×10^9^/l, and there are reports that the use of fedratinib, momelotinib, or low-dose ruxolitinib may be feasible in this setting. It is important to consider the risk of bleeding associated with thrombocytopenia and concomitant use of anticoagulation/antiplatelet therapy, and, possibly, consider prophylaxis with antifibrinolytics. |
| **Reasons provided for scoring the recommendation 6 or less:**  *“Not sure what the term ‘management of spleen’ means... presumably symptomatic splenomegaly. We could continue as in the case with anaemia, ensuring thrombocytopenia is not due to some other cause, eg ITP”* |
| **Q6. Which treatments to increase platelet count can be safely administered to patients  with MF and thrombocytopenia, and when should treatment be initiated/modified?** |
| **CR6:** Treatments to increase platelet counts are rarely effective; agents such as low-dose corticosteroids, danazol, or low-dose immunomodulatory drugs (IMiD^®^) could be considered. There are no data supporting the safety or benefit of thrombopoietin (TPO) mimetics in this setting. Splenectomy can be considered as a last resort in select cases. |
| **Reasons provided for scoring the recommendation 6 or less:**  *“Danazol is not relevant. I also disagree with considering splenectomy for anemia and thrombocytopenia benefit as this is associated with significant morbidity and mortality; it has no proven benefit to improving counts and may actually lead to profound pancytopenia”*  *“Would also consider low dose HMAs in a subset of patients”* |
| **Q7. Aside from access and reimbursement, what factors guide selection of JAK inhibitor therapy in patients with MF and thrombocytopenia?** |
| **CR7:** Factors guiding selection of Janus kinase (JAK) inhibitor monotherapy would include:   - Baseline hemoglobin (Hb) - Degree of thrombocytopenia (eg the only currently approved therapy for patients  with platelets <50×10^9^/l is pacritinib) - MF-related symptoms   For some agents, consideration of drug-specific adverse events (immunosuppression, skin cancer, infection risk, nutritional status, tolerance of gastrointestinal [GI] toxicity, neurotoxicity, cardiovascular adverse events) is also a factor. |
| **Reasons provided for scoring the recommendation 6 or less:**  *“Drug-specific adverse events should be considered for ALL agents as a main factor,  also at lower platelet numbers”* |

GI, gastrointestinal; Hb, hemoglobin; HMA, hypomethylating agent; IMiD, immunomodulatory imide drug; ITP, immune thrombocytopenia; JAK, Janus kinase; MF, myelofibrosis; TPO, thrombopoietin.

**Supplementary Table S9. Voting Round 2: How and When to Determine Prognosis in Patients With MF – verbatim reasons provided for scoring 6 or less on a clinical recommendation**

| **Q13. Which clinical variables are predictive of long-term outcome or survival benefit  in patients receiving JAK inhibitor therapy?** |
| --- |
| **CR13:** The following variables may be useful in predicting the outcome for patients receiving Janus kinase (JAK) inhibitor therapy; however, it is important to consider the limitations of survival data:   - Spleen size reduction (for ruxolitinib, pacritinib, momelotinib, fedratinib, and BMS-911543) - Mutational complexity (for ruxolitinib) - Transfusion independence (for momelotinib) - Anemia response (ruxolitinib, momelotinib, fedratinib, and BMS-911543) - Full blood count (hemoglobin [Hb], platelet count, blasts) - Clonal progression (following discontinuation of ruxolitinib) - Allogeneic stem cell transplantation status (ruxolitinib, momelotinib, fedratinib,  and BMS-911543) |
| **Reasons provided for scoring the recommendation 6 or less:**   - *“Mutational complexity is not a ‘clinical’ variable… Perhaps at the start mention ‘clinical and laboratory’ variables. I’m not sure what is meant by ‘allogeneic stem cell transplantation status’... One reference notes allograft patients responding to [RUX] did better, but this is not reflected in what has been proposed. Perhaps what is meant is ‘maintained (or sustained) response to JAK2 inhibitor prior to allograft’. Include prognostic models, eg MYSEC-PM from the Palandri paper”* - *“We have data that dose of RUX and TI is relevant for OS (RR6 model)”* - *“Non-responding patients are likely a different class of patient who are very likely to have done worse without [RUX] therapy too. In totality, these selected predictive measures have not been independently validated and largely represent post hoc analyses. It is my view that [this] comment here is too weakly supported to be very helpful. Admittedly that eliminates guidance but to my view is the lesser evil”* - *“Not clear how BMT status helps predict response to JAK [inhibitor] therapy”* - *“Not clear what is meant by allograft ‘status’. Does this mean general fitness (eg HCT-CI; Karnofsky)? I have some concern about clonal progression. There is good evidence that certain genomic lesions (eg RAS, TP53) are associated with an adverse outcome, but the clinical meaning of changes in VAF or emergence of other variants (e.g. TET2) is still investigational. I would not want this to be misunderstood by clinicians”* - *“BMS-911543 is not in active clinical development and is irrelevant for these consensus purposes. Also, the anemia response line and full blood counts are unclear, as is the stem cell line”* |

BMT, bone marrow transplantation; Hb, hemoglobin; HCT-CI, Hematopoietic Cell Transplantation-Comorbidity Index; JAK, Janus kinase; MF, myelofibrosis; MYSEC-PM, Myelofibrosis Secondary to PV and ET-Prognostic Model; OS, overall survival; RR6, Ruxolitinib After 6 Months Model;
RUX, ruxolitinib; TI, transfusion independence; VAF, variant allele frequency.

**Supplementary Table S10. Criteria for Ruxolitinib Failure Used in the Re-analysis of the JAKARTA-2, PAC203, and FREEDOM Trials (Adapted from Bose P, Verstovsek S. Hemasphere. 2020; 4[4]:e424)**^1^

| **Relapsed** | Ruxolitinib for ≥3 months with spleen regrowth (defined as <10% SVR or <30% decrease in spleen size by palpation from baseline) following an initial response* |
| --- | --- |
| **Refractory** | Ruxolitinib for ≥3 months with <10% SVR or <30% decrease in spleen size by palpation from baseline |
| **Intolerant** | Ruxolitinib for ≥28 days complicated by development of RBC transfusion requirement (≥2 units/month for two consecutive months); or Grade ≥3 thrombocytopenia, anemia, hematoma/hemorrhage or other, non-hematologic adverse events while on ruxolitinib |

**Response to ruxolitinib is defined as ≥35% reduction in spleen volume from baseline or ≥50% reduction in spleen size for baseline spleen sizes >10cm below left costal margin (LCM), a non-palpable spleen for baseline spleen sizes between 5–10cm below LCM, or not eligible for spleen response for baseline spleen <5cm below LCM. (Harrison CN, et al. Am J Hematol. 2020; 95[6]:594–603)*^2^

*RBC, red blood cell; SVR, spleen volume reduction.*

**Supplementary Table S11. Summary of Prognostication Models Validated in Patients With MF**

The table includes the assigned points for each variable and a description of the risk categories identified with their corresponding median OS, and 95% CI where available

| **Prognostic model** | **When to use score*** | **Patient population included in model validation** | **Variables** | **Points** | **Risk category** | **Score** | **Median OS,  years  (95% CI)** | **Strengths and shortcomings**^3^ |
| --- | --- | --- | --- | --- | --- | --- | --- | --- |
| PMF | | | | | | | | |
| **IPSS**^4^ | Newly diagnosed patients with PMF | - Newly diagnosed PMF (N=1,054) - Median age (range): 64y  (10–90) - Sex, M/F (%): 60.5/39.5 - 7 centers | Age >65y  Hb <10g/dl  WBC >25×10^9^/l  Circulating blasts ≥1%  Constitutional symptoms | 1  1  1  1  1 | Low  Intermediate-1  Intermediate-2  High  / | 0  1  2  3–5  / | 11.3 (9.8, 15.1)  7.9 (6.6, 9.5)  4 (3.6, 4.9)  2.3 (1.9, 2.6)  / | - Uses simple, easy-to-access variables that are routinely collected at diagnosis - Considers few variables, no genetics - ~27 years of observation - Not validated during the course of disease; DIPSS should be used in this setting |
| **DIPSS**^5^ | Patients with PMF  (any stage of disease course, including transplant) | - Newly diagnosed PMF (N=525) - Sex, M/F (%): 63.8/36.2 | Age >65y  Hb <10g/dl  WBC >25×10^9^/l  Circulating blasts ≥1%  Constitutional symptoms | 1  2  1  1  1 | Low  Intermediate-1  Intermediate-2  High  / | 0  1–2  3–4  5–6  / | NR  14.2  4  1.5  / | - Easy-to-access variables - Can be used at any stage during disease course - Focus on anemia - Considers natural history of disease course - 28 years of observation |
| **DIPSS-PLUS**^6^ | Patients with PMF  (any stage of disease course, including transplant) | - Newly diagnosed PMF and follow-up (N=793) - Median age (range): 65y  (14–92) - Sex, M/F (%): 63/37 - 1 center | DIPSS added to:  Unfavorable karyotype  RBC transfusion need  Platelets <100×10^9^/l | 1  1  1 | Low  Intermediate-1  Intermediate-2  High  /  /  /  / | 0  1–2  3–4  4–6  /  /  /  / | 15.4  6.5  2.9  1.3  /  /  /  / | - Easy-to-access variables, except karyotype - Can be used at any stage during disease course - Focus on anemia - Considers natural history of disease course - 39 years of observation |
| **AIPSS-MF**^7^ | Newly diagnosed patients with PMF where NGS/extended genetics are unavailable | - Newly diagnosed PMF (N=1,386) - Median age (range): 69y  (19–94) - Sex, M/F (%): 57.7/42.3 - 60 centers | Sex  Age (y)  Blood blasts (%)  Hb (g/l)  Leukocytes (×10^9^/l)  Platelet count (×10^9^/l)  Constitutional symptoms  Leukoerythroblastosis | - The model was designed to provide personalized predictions of overall survival and leukemia-free survival | | | | - Personalized risk assessment without genomic data - Uses registry-based data - 21 years of observation |
| **MIPSS70+ v2.0**^8^ | Transplant-age patients (≤70y)  with PMF | - Patients ≤70y with PMF (N=311) - Median age; 60y - Sex, M/F (%): 63/37 | VHR karyotype  Unfavorable karyotype  ≥2 HMR mutations  1 HMR mutation  Type 1/like *CALR* absent  Severe anemia (Hb <8g/dl females, Hb <9g/dl males)  Moderate anemia (Hb 8–9.9g/dl females,  Hb 9–10.9 males)  Circulating blasts ≥2%  Constitutional symptoms | 4  3  3  2  2  2  1  1  2 | Very low  Low  Intermediate  High  Very high  /  /  /  / | 0  1–2  3–4  5–8  ≥9  /  /  /  / | NR  16.4  7.7  4.1  1.8  /  /  /  / | - Comprehensive list of parameters included - Variables may not be available for measurement - Considers natural history of disease course |
| **GIPSS**^9^ | Newly diagnosed patients with PMF where NGS/extended genetics are available | - Patients with PMF (N=641) - Median age (range): 63y  (19–89) - Sex, M/F (%): 64/36 - 2 centers | Non-*CALR* mutation type 1  VHR karyotype  Unfavorable karyotype  *ASXL1* mutation  *SRSF2* mutation  *U2AF1* *Q157* mutation | 1  2  1  1  1  1 | Low  Intermediate-1  Intermediate-2  High  /  / | 0  1  2  ≥3  /  / | 26.4  8  4.2  2  /  / | - Only uses genetic parameters to define disease and prognosis - Difficulty obtaining data |
| PMF or post-PV/-ET MF | | | | | | | | |
| **Predictblood**^10^ | Newly diagnosed patients with PMF or first referral | - PMF and post-PV/-ET MF both at diagnosis and during disease course (N=309) | Multistate Cox proportional hazards algorithm incorporating 63 clinical and genomic variables to predict a patient’s probability of transition between stages of disease – namely, chronic-phase disease (ET or PV), advanced-phase disease (MF), AML, and death | Individualized results for:   - Development of MF from ET/PV - Development of AML from either chronic phase or MF (either PMF or secondary MF) - Death (from either chronic phase or MF  [PMF or secondary MF]) - Survival in ET/PV, PMF, and secondary MF | | | | - |
| **RR6 Model**^11^ | Patients with PMF or post-PV/-ET MF, at least 6 months of therapy with ruxolitinib | - PMF and post-ET/-PV patients treated with ruxolitinib for at least 6 months (training cohort, n=209; validation cohort, n=40) - Training cohort   - Median age (range): 67y (37–85)   - Sex, M/F (%): 62.7/37.3 - 17 centers | Ruxolitinib dose <20mg twice daily at baseline, and Months 3 and 6  Palpable spleen length reduction from baseline ≤30% at Months 3 and 6  RBC transfusion need at Months 3 and/or 6  RBC transfusion need at all time points (ie baseline, and Months 3 and 6) | 1  1.5  1  1.5 | Low  Intermediate  High  / | 0  1–2  ≥2.5  / | NR  5.1 (3.6, 6.7)  2.8 (1.8, 4.2)  / | - Can identify patients who may benefit from early treatment switch |
| **MTSS**^12^ | Patients with PMF or post-PV/-ET MF planned for allogeneic stem cell transplantation | - Patients presenting for first allogeneic stem cell transplantation (total cohort, N=361; PMF, n=260;  post-ET/PV, n=101) - Total cohort   - Median age (range): 56y  (18–75)   - Sex, M/F (%): 58/42 - 4 centers | Age >57y  WBC >25×10^9^/l  Platelets <150×10^9^/l  *ASXL1* mutation  Karnofsky performance status <90%  HLA-mismatched unrelated donor  Non *CALR/MPL* driver mutation | 1  1  1  1  1  2  2 | Low  Intermediate  High  Very high  /  /  / | 0–2  3–4  5  6–9  /  /  / | 5y OS, 83%  5y OS, 64%  5y OS, 37%  5y OS, 22%  /  /  / | - Considers transplant-related factors and what to expect; however, transplantation risk may be better determined using a prognostic score that considers the natural history of MF (eg DIPSS, MIPSS70(+), Cambridge score)* |
| Post-PV/-ET MF | | | | | | | | |
| **MYSEC-PM**^13^ | Patients with post-PV and post-ET MF | - Post-PV and post-ET MF at diagnosis (N=685) - Median age (range): 64y  (25–96) - Sex, M/F (%): 52/48 - 16 centers | Hb <11g/dl  Platelets <150×10^9^/l  Circulating blasts ≥3%  *CALR* absent  Constitutional symptoms  Age | 2  1  2  2  1  0.15/y of age | Low  Intermediate-1  Intermediate-2  High  /  / | <11  ≥11–<14  ≥14–<16  ≥16  /  / | NR  9.3 (8.1, NR)  4.4 (3.2, 7.9)  2.0 (1.7, 3.9)  /  / | - Heavily weighted by age and the presence of *JAK2* mutation* - Does not consider additional clinical history that may determine the outcome of MF* - 34 years of observation |

*Consensus expert opinion.

AIPSS-MF, Artificial Intelligence Prognostic Scoring System for Myelofibrosis; AML, acute myeloid leukemia; CI, confidence interval; DIPSS, Dynamic International Prognostic Scoring System; ET, essential thrombocytopenia; GIPSS, Genetically Inspired Prognostic Scoring System; Hb, hemoglobin; HLA, human leukocyte antigen; HMR, high molecular risk; IPSS, International Prognostic Scoring System; M/F, male/female; MF, myelofibrosis; MIPSS70, Mutation-Enhanced International Prognostic Score System for Transplantation-Age Patients With Primary Myelofibrosis; MTSS, Myelofibrosis Transplant Scoring System; MYSEC-PM, Myelofibrosis Secondary to PV and ET-Prognostic Model; NGS, next-generation sequencing; NR, not reached; OS, overall survival; PMF, primary myelofibrosis; PV, polycythemia vera; RBC, red blood cell; RR6, Response to Ruxolitinib After 6 Months; VHR, very high risk; WBC, white blood cell.

**Supplementary References**

1. Bose P, Verstovsek S. JAK inhibition for the treatment of myelofibrosis: Limitations and future perspectives. HemaSphere. 2020; 4(4):e424. doi:10.1097/HS9.0000000000000424

2. Harrison CN, Schaap N, Vannucchi AM, et al. Fedratinib in patients with myelofibrosis previously treated with ruxolitinib: An updated analysis of the JAKARTA2 study using stringent criteria for ruxolitinib failure. Am J Hematol. 2020; 95(6):594–603. doi:10.1002/ajh.25777

3. Duminuco A, Nardo A, Giuffrida G, et al. Myelofibrosis and survival prognostic models: A journey between past and future. J Clin Med. 2023; 12(6):2188. doi:10.3390/jcm12062188

4. Cervantes F, Dupriez B, Pereira A, et al. New prognostic scoring system for primary myelofibrosis based on a study of the International Working Group for Myelofibrosis Research and Treatment. Blood. 2009; 113(13):2895–2901. doi:10.1182/blood-2008-07-170449

5. Passamonti F, Cervantes F, Vannucchi AM, et al. A dynamic prognostic model to predict survival in primary myelofibrosis: A study by the IWG-MRT (International Working Group for Myeloproliferative Neoplasms Research and Treatment). Blood. 2010;115(9):1703–1708. doi:10.1182/blood-2009-09-245837

6. Gangat N, Caramazza D, Vaidya R, et al. DIPSS plus: A refined Dynamic International Prognostic Scoring System for primary myelofibrosis that incorporates prognostic information from karyotype, platelet count, and transfusion status. J Clin Oncol Off J Am Soc Clin Oncol. 2011; 29(4):392–397. doi:10.1200/JCO.2010.32.2446

7. Mosquera-Orgueira A, Pérez-Encinas M, Hernández-Sánchez A, et al. Machine Learning improves risk stratification in myelofibrosis: An analysis of the Spanish Registry of Myelofibrosis. HemaSphere. 2023; 7(1):e818. doi:10.1097/HS9.0000000000000818

8. Tefferi A, Guglielmelli P, Lasho TL, et al. MIPSS70+ Version 2.0: Mutation and Karyotype-Enhanced International Prognostic Scoring System for Primary Myelofibrosis. J Clin Oncol Off J Am Soc Clin Oncol. 2018; 36(17):1769–1770. doi:10.1200/JCO.2018.78.9867

9. Tefferi A, Guglielmelli P, Nicolosi M, et al. GIPSS: Genetically inspired prognostic scoring system for primary myelofibrosis. Leukemia. 2018;32(7):1631–1642. doi:10.1038/s41375-018-0107-z

10. Grinfeld J, Nangalia J, Baxter EJ, et al. Classification and personalized prognosis in myeloproliferative neoplasms. N Engl J Med. 2018; 379(15):1416–1430. doi:10.1056/NEJMoa1716614

11. Maffioli M, Mora B, Ball S, et al. A prognostic model to predict survival after 6 months of ruxolitinib in patients with myelofibrosis. Blood Adv. 2022; 6(6):1855–1864. doi:10.1182/bloodadvances.2021006889

12. Gagelmann N, Ditschkowski M, Bogdanov R, et al. Comprehensive clinical-molecular transplant scoring system for myelofibrosis undergoing stem cell transplantation. Blood. 2019; 133(20):2233–2242. doi:10.1182/blood-2018-12-890889

13. Passamonti F, Giorgino T, Mora B, Guglielmelli P, Rumi E, Maffioli M. A clinical-molecular prognostic model to predict survival in patients with post polycythemia vera and post essential thrombocythemia myelofibrosis. Leukemia. 2017; 31(12):2726–2731.
